# Modeling the clinical and economic impact of universal varicella vaccination in Belgium with dynamic population

**DOI:** 10.1371/journal.pgph.0005636

**Published:** 2026-01-09

**Authors:** John C. Lang, Robert B. Nachbar, Ilaria Xausa, André Bento-Abreu, Barbara Merckx, Manjiri Pawaskar

**Affiliations:** 1 Health Economic and Decision Sciences (HEDS), Biostatistics and Research Decision Sciences (BARDS), Merck Canada Inc., Kirkland, Quebec, Canada; 2 Wolfram Solutions, Wolfram Research, Inc., Champaign, Illinois, United States of America; 3 Market Access, MSD Belgium BVBA, Brussels, VBR, Belgium; 4 Value & Implementation Outcomes Research, Merck & Co., Inc., Rahway, New Jersey, United States of America; PLOS: Public Library of Science, UNITED STATES OF AMERICA

## Abstract

Although universal varicella vaccination (UVV) significantly reduces morbidity and mortality, it has not been implemented in Belgium. We evaluated the clinical and economic outcomes of two-dose UVV in Belgium. A previously published dynamic transmission model with dynamic population age structure was adapted to Belgium. The base case UVV strategy (Strategy 0) consisted of routine two-dose varicella vaccination at ages 1 year (vaccine coverage rate [VCR] = 95%) and 8 years (VCR = 90%), catch-up one-dose varicella vaccination at 8 years (VCR = 70%, 1-year duration), and routine two-dose herpes zoster (HZ) vaccination at 60 years (VCR = 50%). Alternative vaccination strategies were evaluated and reported. The reference strategy consisted of routine two-dose HZ vaccination only. Outcomes were estimated for a 50-year time-horizon. Annual discounting of 3% and 1.5% were applied to costs (in 2023 Euros) and quality-adjusted life-year outcomes, respectively. Under Strategy 0, cumulative varicella incidence, hospitalizations, and deaths decreased by approximately 91%, 89%, and 61% respectively. A transient increase in HZ incidence (that peaked in 2032 at +3% versus the reference strategy) was observed, however, cumulative HZ cases decreased by 3% over 50 years. Under the payer perspective, Strategy 0 had an incremental cost-effectiveness ratio of €11,260. Under the societal perspective, Strategy 0 resulted in cost savings, with total costs decreasing by €17,524,487. UVV can significantly reduce the burden of varicella with marginal impact on HZ cases and be cost-effective in Belgium versus routine HZ vaccination only.

## Introduction

Varicella (chickenpox) is a highly contagious disease caused by the varicella zoster virus (VZV) [1]. Following primary infection, VZV may reactivate later in life resulting in herpes zoster (HZ) [1,2]. Although varicella is usually mild, severe complications are possible and include hospitalization, and, in rare cases, death, particularly in neonates, immunocompromised persons, and adults [3,4]. Globally, there are over 4 million varicella-related hospitalizations and approximately 4,200 varicella-related deaths, annually [3]. In Belgium, the annual incidence of general practitioner visits was estimated to be 346 and 378 per 100,000 for chicken pox and herpes zoster, respectively; hospitalization rates were 5.3 and 14.2 per 100,000 [5].

Varicella-containing vaccines are available as a monovalent vaccine that protects against varicella (e.g., VARIVAX [V-MSD] by Merck Sharp & Dohme LLC, Rahway, NJ, USA [MSD] and VARILRIX [V-GSK] by GlaxoSmithKline SA, Belgium [GSK]) or as a quadrivalent vaccine that also protects against measles, mumps, and rubella (MMR) (e.g., ProQuad [MMRV-MSD] by MSD or PRIORIX TETRA [MMRV-GSK] by GSK). Vaccination against HZ (Shingrix [HZ-GSK] by GSK) is available in Belgium as a two-dose series for older and/or immunocompromised adults. Universal varicella vaccination (UVV) programs have been implemented in many countries [6], including the US, Italy, Germany, Spain, and Israel, resulting in significant reductions in clinical and economic burden of varicella [3,4,7–10]. Although MMR vaccination is currently included in the Belgian national immunization program (NIP) at 1 year old and 8 years old, varicella vaccination is not, primarily due to concerns that potential increases in HZ incidence may preclude the cost-effectiveness of proposed UVV programs [11]. Bilcke and colleagues concluded that the cost-effectiveness of implementing a UVV program in Belgium was unclear and was strongly influenced by assumptions about exogenous boosting and the time horizon [12,13].

According to the exogenous boosting hypothesis, exposure to VZV increases resistance to reactivation as HZ [14]. Hence, the incidence of HZ may increase following the introduction of UVV. Since HZ cases are generally more severe than varicella cases, the decision to introduce a UVV program may be subject to an intergenerational conflict of interest [15,16]. Thus, the full impact of the potential UVV program must be evaluated with contemporary data, including recent real-world evidence from the US, where 25 years of experience with UVV showed no impact on HZ incidence [17–19].

Multiple exogenous boosting modelling frameworks have been proposed, including permanent full immunity, temporary full immunity, and progressive immunity [14]. Additionally, VZV dynamic transmission models (DTMs) may also include detailed demographic modelling, especially for populations with rapidly changing demographics, to more accurately account for the full effects of UVV on HZ due to population ageing [20]. However, cost-effective evaluations that account for all the above factors are rare. A 2023 systematic literature review found that, of 34 DTMs that evaluated the impact of UVV, only 11 (11/34) included the effects of UVV on HZ and only 2 (2/11) modeled changes in demographic structure over time [21].

The objective of this study was to evaluate the cost-effectiveness of 2-dose UVV strategies in Belgium by estimating the long-term clinical and economic impact on varicella infection and HZ reactivation using a DTM implementing dynamic demographics and exogenous boosting.

## Materials and methods

### Dynamic transmission model

A deterministic age-structured DTM consisting of a system of ordinary differential equations (ODEs) and following a Maternal-Susceptible-Exposed-Infectious-Recovered structure, including dynamic demographics and temporary full immunity exogenous boosting, was previously developed to evaluate UVV programs in the United Kingdom [22]. The DTM was implemented with the mechanism of temporary full immunity for exogenous boosting due to it being a parsimonious and well-established mechanism within the current literature [14]. This model was adapted to Belgium and parameterized with country-specific demographic, epidemiological, and healthcare resource use data (see Tables A-B and Sections A-B and D in S1 File; Tables A-U in S1 File; and Figs A-C in S1 File). The model was calibrated with country-specific varicella seroprevalence [23] and herpes zoster incidence [5,24] data (see Section C in S1 File; Tables J-K in S1 File). Calibration was implemented using a maximum likelihood objective function in three steps, as previously detailed by Sharomi and colleagues [22]. Briefly, the VZV transmission parameters were first calibrated to seroprevalence data using predefined values for HZ reactivation parameters. Second, HZ reactivation parameters were calibrated to HZ incidence data using the VZV transmission parameters obtained from the first step. Finally, VZV transmission parameters were re-calibrated using the values for HZ reactivation parameters obtained from the second step. This final step was required, as it was assumed that persons experiencing HZ were infectious. Varicella and herpes zoster deaths were computed using reported case fatality ratios, and hence, deaths were proportional to the number of cases (see Section B.2 in S1 File). The model was simulated using Mathematica 13.1 (Tables 1 and 2).

**Table 1:** Varicella resource use and cost parameters (costs reported in 2023 Euros).

Cost Parameter	Age (years)							Reference
	<5	5-10	10-15	15-20	20-60	60-65	≥65	
**Direct varicella treatment costs**								
Fraction of varicella cases seeking GP care	30.8%	24.4%	66.9%	28.6%	40.8%	39.5%		[25]
Varicella GP consultation cost	€ 44.25							[5]^A^
Fraction of varicella cases requiring hospitalization	0.54%	0.07%	0.53%	0.68%	1.67%	1.32%		[25]
Varicella hospitalization cost	€ 3,538.29							[5]^A^
Total direct varicella treatment cost per varicella infection^C^	€ 32.73	€ 13.36	€ 48.29	€ 36.80	€ 77.05	€ 64.03		Calculated^B^
**Productivity loss due to varicella infections (indirect varicella costs)**								
Average daily wage	€ 212.02							[26]
Workdays lost due to varicella infection	0.6			5.7			0	[13,27–29]
Workdays lost due to varicella hospitalization	5.1						0	[13]
Total indirect varicella cost per varicella infection^D^	€ 44.96	€ 31.80	€ 90.78	€ 353.40	€ 511.38	€ 491.18	€ 0.00	Calculated^B^

^A^Inflated to 2023 Euros

^B^For details see Section D in S1 File.

^C^Total direct cost per varicella case is equal to (fraction seeking GP) x (GP cost) + (fraction requiring hospitalization) x (hospitalization cost). Note: that all values shown are rounded to two decimal places.

^D^Total indirect cost per varicella case is equal to (daily wage x ((fraction seeking GP) x (workdays lost due to varicella infection) + (fraction requiring hospitalization) x (workdays lost due to varicella hospitalization)). Note: that all values shown are rounded to two decimal places.

**Table 2:** HZ resource use and cost parameters (costs reported in 2023 Euros).

Cost Parameter	Age (years)										Reference
	<5	5-10	20-40	40-50	50-60	60-65	65-70	70-80	80-90	≥90	
**Direct HZ treatment costs**											
Fraction of HZ cases seeking GP care	96.6%	99.2%	98.8%	98.3%	97.9%	97.0%		95.6%	92.1%	94.8%	[5]^A^
HZ GP consultation cost	€ 67.17				€ 107.57	€ 121.04		€ 153.42	€ 168.62	€ 164.28	Calculated^B^
Fraction of HZ cases requiring hospitalization	3.4%	0.8%	1.2%	1.7%	2.1%	3.0%		4.4%	8.0%	5.2%	[5]
HZ hospitalization cost	€ 5,767.22				€ 5,889.52	€ 5,930.28		€ 5,996.58	€ 6,043.93	€ 6,030.40	Calculated^B^
Total direct HZ treatment cost per HZ episode^C^	€ 260.81	€ 115.63	€ 137.98	€ 165.66	€ 230.36	€ 293.60		€ 408.16	€ 635.77	€ 470.89	Calculated^B^
**Productivity loss due to HZ episodes (indirect HZ costs)**											
Workdays lost due to HZ episode	3.3						0.0				[30]
Workdays lost due to HZ hospitalization	7.0						0.0				[5]
Total indirect HZ cost per HZ episode^D^	€ 735.03	€ 715.28	€ 718.32	€ 722.09	€ 725.16	€ 731.72	€ 0.00				Calculated^B^

^A^We assume that all HZ cases seek either GP care or require hospitalization.

^B^For details see Section D in S1 File.

^C^Total direct cost per HZ case is equal to (fraction seeking GP) x (GP cost) + (fraction requiring hospitalization) x (hospitalization cost). Note: that all values shown are rounded to two decimal places.

^D^Total indirect cost per HZ case is equal to (daily wage x ((fraction seeking GP) x (workdays lost due to HZ episode) + (fraction requiring hospitalization) x (workdays lost due to HZ hospitalization)). Note: that all values shown are rounded to two decimal places.

### Dynamic demographic model

This DTM uses a dynamic demographic model that combines historical demographic data with projections to simulate the changing demographics of the population over the time horizon. Specifically, the model incorporated population [31], mortality [32], and fertility data [33] (stratified by age and by year) and migration data [34] (stratified by age, but assumed constant over time).

### Exogenous boosting assumptions

In previous VZV DTMs, most commonly, exogenous boosting is modeled as either assuming temporary full immunity or progressive immunity [14]. Progressive immunity assumes that exogenous boosting grants partial immunity to HZ that wanes over time; hence, immunity is progressive and subsequent episodes of exogenous boosting result in increasing levels of partial immunity to HZ. In contrast, we adopted a temporary full immunity approach, which assumes that exogenous boosting grants full immunity to HZ that wanes over time. Specifically, the likelihood of an exposure to a varicella-infected individual resulting in exogenous boosting and the average duration of full protection from exogenous boosting were estimated to be 33.45% and 81.3 years, respectively [22].

### Vaccine properties

Vaccine properties and parameterization for monovalent and quadrivalent varicella vaccines (V-MSD, V-GSK, MMRV-MSD, MMRV-GSK) in the context of this DTM have been described previously [22,35] (Table 3). Briefly, varicella vaccination may fail to produce an immune response (vaccine failure), or may result in temporary or lifetime immunity to varicella infection, based on vaccine take. Vaccination against HZ (HZ-GSK) was assumed to confer temporary full immunity with exponential waning. The exponential waning rate, ωz, was estimated through calibration against previously reported HZ vaccine efficacy data [36] (Section E in S1 File; Table V in S1 File; Figs D-E in S1 File).

**Table 3:** Vaccine properties.

Parameter	V-MSD and MMRV-MSD	V-GSK and MMRV-GSK	Source
Vaccine failure rate	4%	5%	[37,38]
Vaccine take rate^A^			
First dose	90.3%	61.7%	[35]
Second dose	69.0%	83.4%	[35]
Overall	97.0%	93.8%	[35]
Duration of protection (temporary immunity to varicella infection)	1.2 years	0.9 years	[35]

^A^Vaccine take is defined as the proportion of seroconverted individuals (i.e., those in whom the vaccine does not fail) for whom the vaccine confers permanent protection. For example, 96% of individuals vaccinated with V-MSD seroconvert, and thus 96% x 90.3% = 86.7% of individuals vaccinated with V-MSD are estimated to develop permanent immunity.

### Vaccination strategies

We assumed a reference strategy comprising only routine HZ vaccination strategy (two-dose HZ vaccination at 60 years old with 50% vaccine coverage rate [VCR]). Vaccination Strategies 0–3 are summarized in Table 4. Our base case vaccination strategy (Strategy 0) consisted of routine two-dose UVV with MMRV-MSD at ages 1 year (vaccine coverage rate [VCR] = 95%) and 8 years (VCR = 90%), and catch-up one-dose vaccination with MMRV-MSD at age 8 years (VCR = 70%, 1-year program duration). This was chosen to align with the current vaccination schedule for MMR in Belgium. The base case strategy also included routine two-dose vaccination with HZ-GSK at age 60 (VCR = 50%). In addition, we considered three variations of the base case strategy. Strategy 1 assumed lower VCR for routine varicella vaccination (first-dose VCR: 90%; second-dose VCR = 85%). Strategy 2 administered varicella catch-up vaccination to 5-year-olds using the monovalent V-MSD vaccine formulation (given the current NIP vaccination schedule co-administration of MMR and varicella vaccination is not possible for 5-year-olds). Strategy 3 included an additional two-dose catch-up vaccination program with HZ-GSK for 60–85-year-olds (VCR = 50%, 1-year program duration). Nine additional vaccination strategies were included as scenario analyses. Eight scenarios (Strategies 4–11) are variations on the base case strategy. For completeness, one strategy (Strategy 12) included routine two-dose HZ vaccination for 60-year-olds (VCR = 50%) and catch-up two-dose HZ vaccination for 60–85-year-olds (VCR = 50%, 1-year program duration). For additional details, see Section F in S1 File (Table W in S1 File).

**Table 4:** Varicella vaccine strategies.

Strategy	Vaccination Age (years)					Vaccination Coverage				
	Varicella^A^			Herpes Zoster		Varicella^A^			Herpes Zoster	
	Primary		Booster	Routine	Catch-up^B^	Primary		Booster	Routine	Catch-up^B^
	Routine	Catch-up^B^	Routine			Routine	Catch-up^B^	Routine		
Reference	N/A	N/A	N/A	60	N/A	N/A	N/A	N/A	50%	N/A
0 (Base case)	1	8	8	60	N/A	95%	70%	90%	50%	N/A
1	1	8	8	60	N/A	90%	70%	85%	50%	N/A
2	1	5	8	60	N/A	95%	70%	90%	50%	N/A
3	1	8	8	60	60-85	95%	70%	90%	50%	50%

^A^MMRV-MSD vaccines were administered to 1- and 8-year-olds. V-MSD vaccines were administered to 5-year-olds. MMRV vaccines were assumed to replace MMR vaccines.

^B^Catch-up programs were assumed to have a duration of 1 year.

### Model outcomes and cost-effectiveness analysis

All outcomes were evaluated over a 50-year time-horizon (2023–2072). Clinical outcomes included varicella cases, HZ cases, and associated outpatient visits, hospitalizations, and deaths. Quality-adjusted life-year (QALY) loss due to varicella and HZ outcomes were also reported. Economic outcomes were evaluated from the payer (direct cost only) and societal (direct and indirect cost) perspectives. Direct costs comprised outpatient and hospitalization treatment costs for varicella and HZ, as well as vaccination acquisition and administration costs. Indirect costs comprised productivity loss of patients (for cases in adults) or their caregivers (for cases in children) due to varicella and HZ, employing a human capital approach. Indirect costs associated with vaccine administration were not included in this analysis and we assumed that no productivity loss occurred for individuals aged 65 years and older. Costs were adjusted for inflation and reported in 2023 Euros. Costs and QALYs were discounted at 3% and 1.5%, respectively [39]. Cost-effectiveness was evaluated using both incremental cost-effective ratio (ICER)


$$ICER=\frac{Incremental\;costs}{Incremental\;QALYs}$$

and net monetary benefit


NMB=(Incremental QALYs)×WTP−(Incremental costs),

where WTP is the willingness-to-pay threshold of €40,000 [40].

### Sensitivity analysis

Parameter uncertainty was evaluated through probabilistic (PSA) and one-way deterministic sensitivity analysis (DSA). PSA distributions for calibration parameters were estimated during the calibration procedure [22]. PSA distributions for non-calibration parameters are recorded in Section G in S1 File (Table X in S1 File). The PSA evaluated 1,024 realizations of parameter sets drawn from the above distributions using a Latin Hypercube Sampling (LHS) approach (Section G in S1 File; Table X in S1 File) [41]. The parameter ranges for DSA were the 95% quantile intervals estimated from the PSA parameter distributions. For the DSA, the model was evaluated by varying one parameter at a time at the lower and upper bounds identified for the DSA.

## Results

Model calibration results are displayed in Section H.1 in S1 File (Figs F-I in S1 File).

### Clinical outcomes

**Fig 1:**
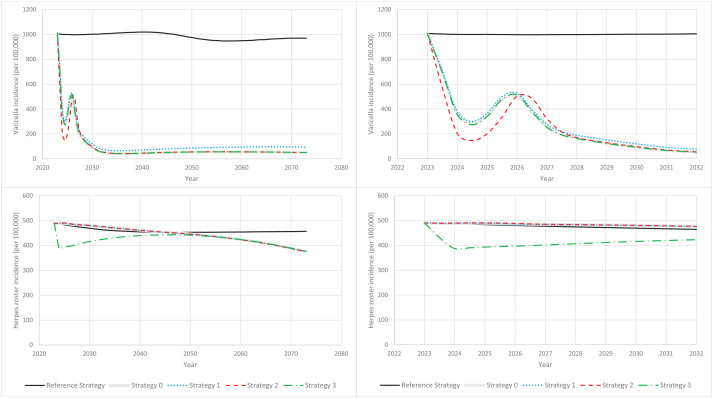
Varicella and herpes zoster incidence versus time (2023–2072). (Solid black line) HZ-vaccination-only reference strategy. (Solid grey line) Base case vaccination strategy (Strategy 0). (Dotted blue line) Strategy 1: Lower first- and second-dose VCR. (Dashed red line) Strategy 2: Earlier first-dose varicella catchup. (Dash-dotted green line) Strategy 3: With both routine and catch-up herpes zoster vaccination. (Top panels) Varicella incidence per 100,000 versus time. (Bottom panels) Herpes zoster incidence per 100,000 versus time. (Left panels) Time-horizon: 2023-2072. (Right panels) Time-horizon: 2023–2032.

In the absence of UVV (reference strategy, with routine HZ vaccination only), annual varicella incidence decreased by approximately 4%, from 1,008 per 100,000–970 per 100,000, over the 50-year time-horizon (Fig 1, top panel). Annual HZ incidence decreased by approximately 7%, from 490 per 100,000–457 per 100,000 (Fig 1, bottom panel). Cumulatively, over the 50-year time-horizon we estimated 6,525,904 varicella cases, 1,905,853 outpatient cases, 27,924 hospitalizations, and 61 deaths. Cumulative HZ cases and deaths were estimated to be 3,044,798 and 467, respectively. We estimated a total cumulative QALY loss of 81,204, and total cumulative payer and societal costs of €1,253,338,674 and €2,099,961,167, respectively.

**Fig 2:**
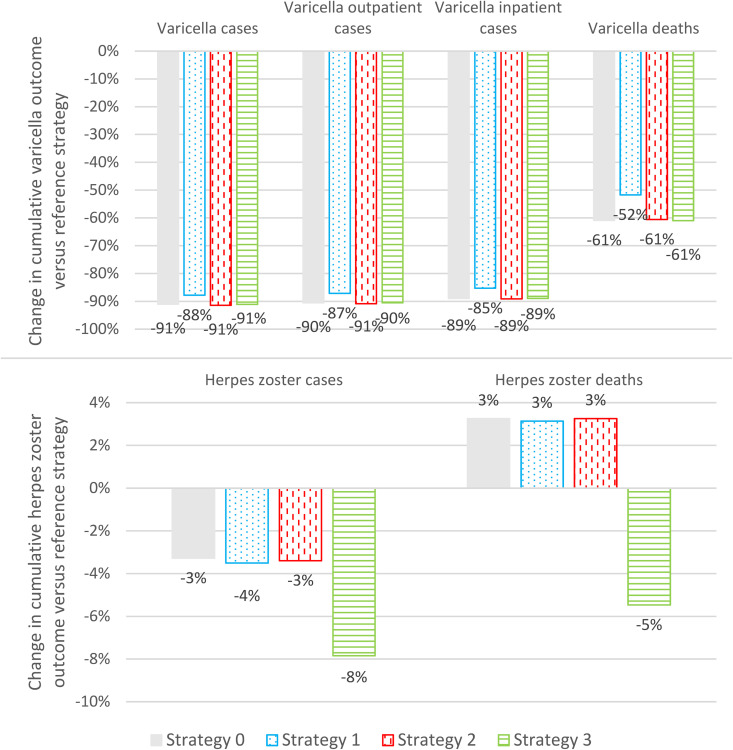
Change in cumulative varicella and herpes zoster clinical outcomes versus reference strategy (2023-2072). (Grey) Strategy 0: Base case vaccination strategy. (Blue dotted) Strategy 1: Lower first- and second-dose vaccine coverage rates. (Red vertical dash) Strategy 2: Earlier first-dose varicella catch-up. (Green horizonal lines) Strategy 3: With both routine and catch-up herpes zoster vaccination.

**Fig 3:**
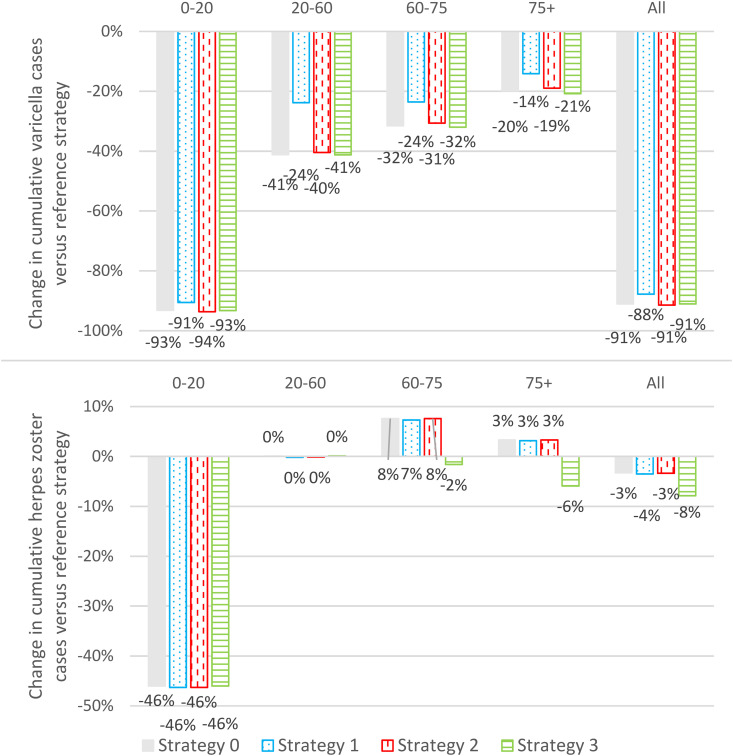
Change in cumulative varicella and herpes zoster cases stratified by age versus reference strategy (<20 years, 20-60 years, 60-75 years, and ≥75 years). (Grey) Strategy 0: Base case vaccination strategy. (Blue dotted) Strategy 1: Lower first- and second-dose vaccine coverage rates. (Red vertical dash) Strategy 2: Earlier first-dose varicella catch-up. (Green horizontal lines) Strategy 3: With both routine and catch-up herpes zoster vaccination.

The impact of UVV on varicella outcomes was similar across strategies. Strategies 0–3 decreased annual varicella incidence by 90–95% over the 50-year time-horizon with respect to the reference strategy (Fig 1, top panel). UVV strategies reduced cumulative varicella cases, varicella outpatient cases, and varicella hospitalizations by 85–90%, and varicella deaths by 50–60% (Fig 2, top panel). Cumulative varicella cases were reduced in all age groups (<20 years, 20–60 years, 60–75 years, and ≥75 years) (Fig 3, top panel).

The impact of UVV on HZ outcomes depended on whether catch-up HZ vaccination was included (Strategy 3) or not (Strategies 0–2). For Strategy 3, HZ incidence was at least 2% lower than the reference strategy over the entire time horizon and 18% lower than the reference strategy at 50 years (Fig 1, bottom panel). Overall, cumulative HZ cases and deaths were reduced by 8% and 5%, respectively (Fig 2, bottom panel). Cumulative HZ cases were reduced in all age groups (Fig 3, bottom panel). For Strategies 0–2, there was a transient increase in HZ incidence that peaked in 2032 at 2–3% higher than the reference strategy; by the end of the time horizon HZ incidence was approximately 18% lower than the reference strategy (Fig 1, bottom panel). Overall, Strategies 0–2 reduced cumulative HZ cases by 3–4%, but increased cumulative HZ deaths by 3%, with approximately 15 additional deaths over 50 years (Fig 2, bottom panel). Cumulative HZ cases were 46% lower in <20-year-olds and 3–8% greater in ≥60-year-olds (Fig 3, bottom panel).

### Economic outcomes and cost-effectiveness analysis

Incremental cumulative cost and QALY outcomes for all strategies are displayed in Table 5 and in Section H.2 in S1 File (Tables Y-Z in S1 File). A further breakdown of incremental QALYs by cause (i.e., varicella disease, varicella deaths, herpes zoster disease, and herpes zoster deaths) is presented in Table 6. The year in which each strategy’s net monetary benefit (relative to the reference strategy, with a WTP of €40,000) becomes positive is presented in Table 7. For Strategies 0–2, QALYs were gained from varicella disease and deaths and QALYs were lost from herpes zoster disease and deaths. For Strategy 3, QALYs were gained from all causes. For all strategies, there was a net gain in QALYs. Under the payer perspective, the cost-effectiveness frontier consisted of the reference strategy and Strategies 1–3; Strategy 0 was weakly dominated. Under the societal perspective, Strategies 0–2 were cost-saving relative to the reference strategy and the cost-effectiveness frontier consisted of Strategies 1–3. Under both perspectives, Strategy 2 (early first-dose varicella catch-up) was the most cost-effective strategy at a WTP of €40,000. Equivalently, Strategy 2 had the highest net monetary benefit (NMB), approximately €436 million and €625 million under the payer and societal perspectives, respectively. Under both perspectives the NMB for Strategy 1 was approximately 1% less than Strategy 2 and the NMB for Strategy 0 was approximately 2–3% less than Strategy 2. In contrast, the NMB for Strategy 3 was significantly less than for Strategy 2 (27% and 17% less under the payer and societal perspectives, respectively) due to the inclusion of the HZ catch-up vaccination program. Strategy 3 also had the highest incremental ICER under both payer and societal perspectives, at €61,780 and €60,452, respectively, exceeding the WTP of €40,000 [40]. Inclusion of strategies for scenario analysis (see Section F in S1 File, Table W in S1 File; and also Section H.2 in S1 File, Tables Y-Z in S1 File) had no impact on the cost-effectiveness frontier or on the ranking of the three most cost-effective strategies.

**Table 5:** Incremental costs, incremental quality-adjusted life-years (QALYs), and frontier analysis.

Strategy	Incremental QALYs^A^	Incremental costs^B^ (millions)	Incremental cost-effectiveness ratio (ICER)^B^		Net monetary benefit^B^ (millions)
			ICER (vs reference)	Incremental ICER^C^	
**Payer Perspective**					
Reference strategy^C^ (routine HZ vaccination only)	0 (N/A)	€ 0 (N/A)	N/A (N/A)		€ 0 (N/A)
Strategy 1^C^	14,602 (8,245, 21,585)	€ 156.5 (€ 53.2, € 257.3)	€ 10,721 (€ 3,851, € 20,634)	€ 10,721 (€ 3,851, € 20,634)	€ 427.5 (€ 185.8, € 712.2)
Strategy 0	15,035 (8,979, 22,578)	€ 169.3 (€ 63.3, € 274.7)	€ 11,260 (€ 4,19, € 21,116)	Weakly dominated (N/A)	€ 432.1 (€ 186.3, € 721.7)
Strategy 2^C^	15,154 (8,625, 22,280)	€ 170.1 (€ 72.2, € 273.5)	€ 11,227 (€ 4,826, € 21,498)	€ 24,630 (Cost-saving, € 228,642)	€ 436.0 (€ 188.5, € 729.9)
Strategy 3^C^	20,508 (13,171, 28,363)	€ 500.9 (€ 363.6, € 647.9)	€ 24,424 (€ 14,917, € 41,095)	€ 61,780 (€ 26,29, € 220,826)	€ 319.4 (-€ 11.1, € 667.5)
**Societal Perspective**					
Reference strategy^C^ (routine HZ vaccination only)	0 (N/A)	€ 0 (N/A)	N/A (N/A)		€ 0 (N/A)
Strategy 1^C^	14,602	-€ 21.2 (-€ 167.1, € 102.2)	Cost-saving (N/A)	Cost-saving (N/A)	€ 605.4 (€ 331.5, € 917.4)
Strategy 0	15,035 (8,979, 22,578)	-€ 17.5 (-€ 158.5, € 113.5)	Cost-saving (N/A)	Weakly dominated (N/A)	€ 618.9 (€ 334.8, € 932.1)
Strategy 2^C^	15,154 (8,625, 22,280)	-€ 18.4 (-€ 154.5, € 110.8)	Cost-saving (N/A)	€ 5,045 (Cost-saving, € 320,826)	€ 624.6 (€ 337.8, € 938.5)
Strategy 3^C^	20,508 (13,171, 28,363)	€ 305.2 (€ 123.3, € 474.0)	€ 14,882 (€ 5,108, € 30,716)	€ 60,452 (€ 26,262, € 227,539)	€ 515.1 (€ 134.2, € 916.2)

^A^Incremental QALYs were the same under both payer and societal perspectives.

^B^Values in parentheses indicate 2.5% and 97.5% quantiles of the PSA realizations.

^C^Strategies 1, 2, 3, and the reference strategy are on the cost-effectiveness frontier: the incremental ICER for Strategy 1 is computed versus the reference strategy, the incremental ICER for Strategy 2 is computed versus Strategy 1, and the incremental ICER for Strategy 3 is computed versus Strategy 2.

**Table 6:** Incremental quality-adjusted life-years (QALYs) by cause (varicella disease, varicella deaths, herpes zoster disease, and herpes zoster deaths).

Strategy	Varicella		Herpes Zoster		Total
	Disease	Deaths	Disease	Deaths	
Reference strategy	0	0	0	0	0
Strategy 0	16,648	583	-2,126	-70	15,035
Strategy 1	16,132	547	-2,008	-68	14,602
Strategy 2	16,762	586	-2,123	-71	15,154
Strategy 3	16,655	586	3,074	193	20,508

**Table 7:** Year when net monetary benefit (relative to reference strategy, a with willingness-to-pay of €40,000) becomes positive.

Strategy	Year	
	Payer Perspective	Societal Perspective
Reference strategy	N/A	
Strategy 0	2023	
Strategy 1	2023	
Strategy 2	2023	
Strategy 3	2039	2035

### Sensitivity analysis

**Fig 4:**
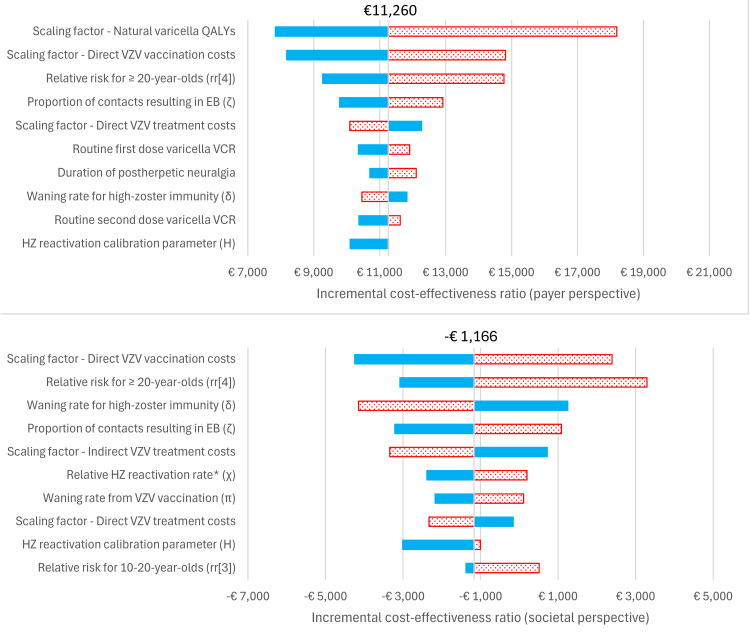
Tornado diagram for Strategy 0 incremental cost-effectiveness deterministic sensitivity analysis (DSA) results. (Solid blue bars) Parameter lower bound. (Dotted red bars) Parameter upper bound. (Top) Payer perspective. (Bottom) Societal perspective. EB: exogenous boosting; HZ: herpes zoster; QALY: quality-adjusted life-year; VCR: vaccine coverage rate; VZV: varicella zoster virus. * Relative reactivation rate for VZV and HZ vaccinated individuals with respect to reactivation rate of individuals who were infected with natural varicella.

**Fig 5:**
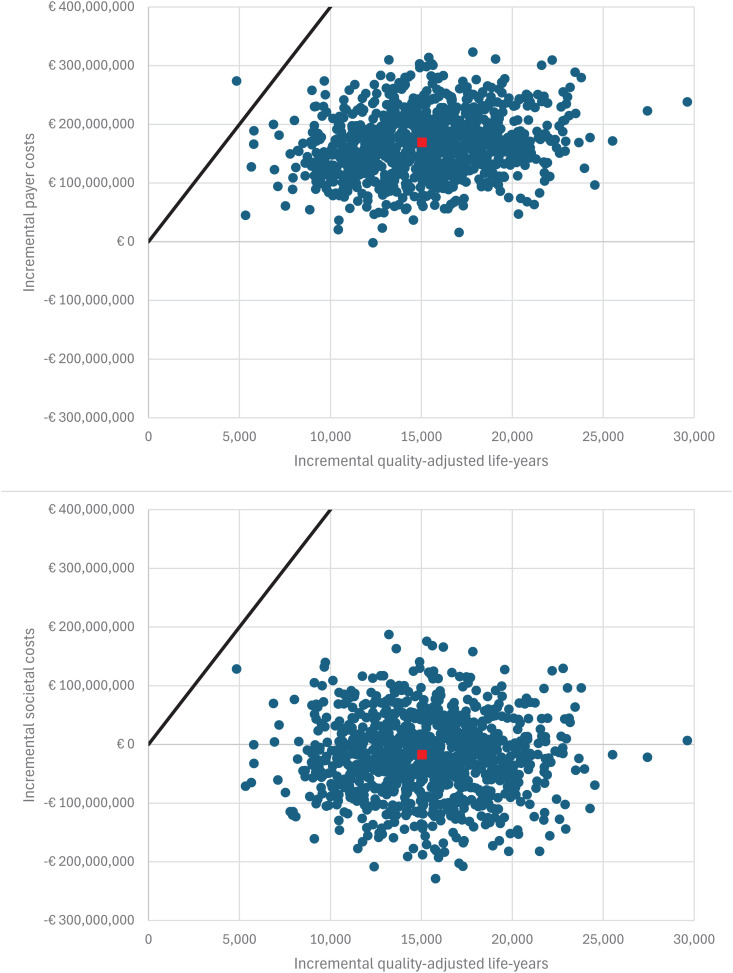
Incremental cost versus incremental QALY scatter plot for Strategy 0 probabilistic sensitivity analysis (PSA) results. (Square) Model outcome with base case values. (Circles) Probabilistic sensitivity analysis realizations. (Line) Willingness-to-pay threshold.

**Fig 6:**
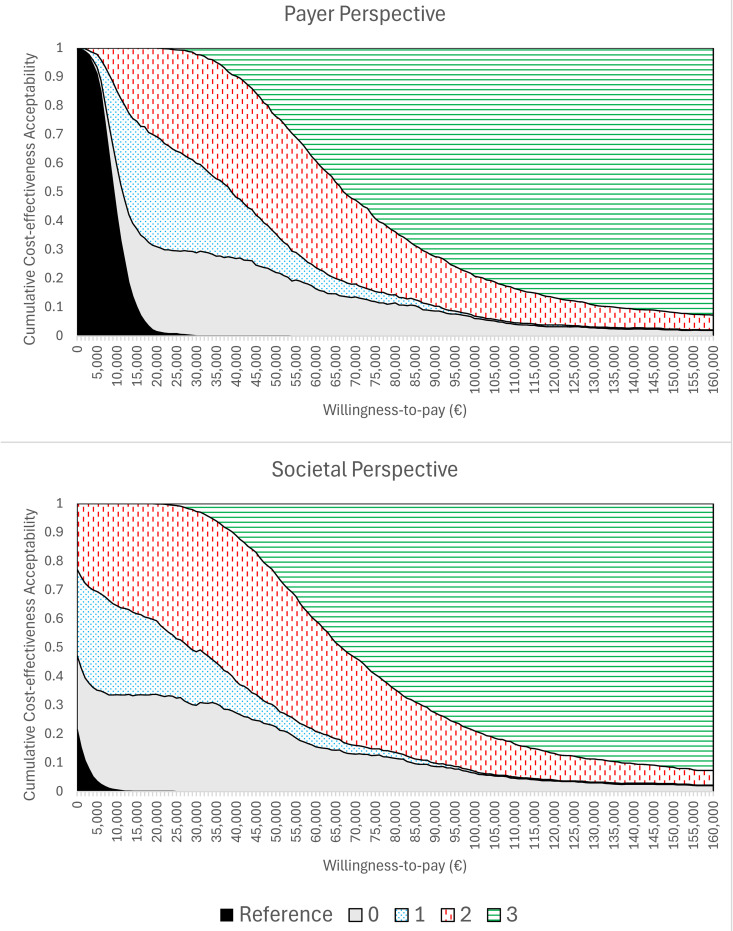
Cumulative cost-effectiveness acceptability curve for the reference strategy and Strategies 0-3. Fraction of PSA realizations where (black) the reference strategy was cost-effective, (gray) Strategy 0 was cost-effective, (blue dotted) Strategy 1 was cost-effective, (red vertical dash) Strategy 2 was cost-effective, and (green horizontal lines) Strategy 3 was cost-effective. (Top) Payer perspective. (Bottom) Societal perspective.

In the DSA the five most impactful model parameters for Strategy 0 (base case) under the payer perspective were: the scaling factor for QALYs for individuals infected with natural varicella, the scaling factor for direct varicella vaccination costs, the relative risk of infection for over-20-year-olds, the proportion of contacts with varicella-infected individuals that result in exogenous boosting, and the scaling factor for direct varicella treatment costs (Fig 4). Under the societal perspective the five most impactful model parameters for Strategy 0 were: the scaling factor for direct varicella vaccination costs, the relative risk of infection for over-20-year-olds, the waning rate for high-zoster immunity (following natural varicella infection), the proportion of contacts with varicella-infected individuals that result in exogenous boosting, and the scaling factor for indirect varicella treatment costs. Results were qualitatively similar for Strategies 1–3 (see Section H.3 in S1 File; Figs J-L in S1 File). For Strategies 0–2, 99.9% and 100.0% of PSA realizations were cost-effective at a WTP of € 40,000 under the payer and societal perspectives, respectively. Under the societal perspective, 63.9-66.0% of PSA realizations were cost-saving for Strategies 0–2 (see Fig 5 and Section H.3 in S1 File; Figs M-O in S1 File). For Strategy 3, 97.1% and 99.9% of PSA realizations were cost-effective under the payer and societal perspectives, respectively, and two realizations (0.2%) were cost-saving. A cost-effectiveness acceptability curve is included in Fig 6. At a willingness-to-pay threshold of €40,000, Strategy 2 had the highest NMB in 42.0% and 50.6% of PSA realizations under the payer and societal perspectives, respectively. For comparison, the highest NMB occurred for Strategy 0 in 26.8% and 27.3% of PSA realizations, for Strategy 1 in 21.3% and 11.0% of PSA realizations, for Strategy 3 in 9.8% and 11.0% of PSA realizations, and for the reference strategy in 0.1% and 0.0% of PSA realizations. These results were consistent with the result that the NMB was similar between Strategies 0–1, lower for Strategy 3, and lowest for the reference strategy (Table 5).

## Discussion

We adapted an age-structured DTM for varicella zoster virus to Belgium to evaluate the cost-effectiveness of different UVV strategies over a 50-year time-horizon, implementing a temporary full immunity exogenous boosting model structure to account for the impact of UVV on HZ outcomes. The DTM also accounted for changing demographics in Belgium via a dynamic demographic model. Overall, assuming a WTP threshold of €40,000, we found that all UVVs were cost-effective with respect to the reference strategy (where only HZ vaccination was considered). These results were robust to sensitivity analyses. In other words, in the absence of UVV (i.e., in the reference scenario), nearly the entire population acquired varicella at some point during their lives. Thus, although the average varicella infection had a small health impact, the population-level aggregate impact of varicella was sufficient to justify a UVV program.

The base case strategy (Strategy 0) resulted in a substantial reduction in the clinical burden of varicella (91% of varicella cases, 89% hospitalizations, and 61% deaths). Strategy 0 resulted in an overall 3% decrease in cumulative HZ cases, however, a small transient increase in annual HZ incidence (peaking at +3% over the reference strategy) and a small increase in the cumulative number of HZ-related deaths (approximately 0.3 additional deaths per year) were observed. Given that the case fatality rate for HZ cases was approximately 0 for <60-year-olds, 2 per 100,000 for 60–75-year-olds, and 45–90 per 100,000 for ≥75-year-olds, even a small increase in the age of onset of HZ (see Fig 3) can have a significant impact on the number of HZ deaths predicted by the model, and thus the increase in HZ-related deaths was most likely a result of the moderate age-shift observed in HZ cases in ≥60-years-olds. Whether this effect is a limitation of the age stratification of the model and availability/accuracy of age-stratified HZ case fatality data, or whether this phenomenon is consistent with real-world evidence is an important subject of future research. Under both the payer and societal perspectives, Strategy 0 was the second most cost-effective UVV strategy (WTP = €40,000) with a NMB that was 0.9% less than Strategy 2 (the UVV strategy with early first-dose varicella catch-up vaccination). The improvement in outcomes for Strategy 2 versus Strategy 0 was the result of the indirect effects of catchup vaccination for 5-year-olds (Strategy 2) versus 8-year-olds (Strategy 0). Prior to universal varicella vaccination, approximately 68.8% of children had contracted varicella by age 5 years versus 91.5% of children who had contracted varicella by age 8 years (Fig H in S1 File). As only varicella-naïve children were eligible for vaccination, more children were vaccinated under Strategy 2 than Strategy 0 and more varicella cases were averted due to the direct effects of varicella vaccination. As the risk profiles of 8-year-olds and 5-year-olds were similar, the cost-effectiveness of Strategy 2 versus Strategy 0 was not significantly affected by these direct effects (e.g., for a static model with constant force of infection the cost-effectiveness can generally be expected to be independent of the treatment uptake). However, because the catchup program under Strategy 2 vaccinated more susceptible children than Strategy 0, the indirect effects of vaccination were more pronounced under Strategy 2 than under Strategy 0. Thus, the initial decline in varicella incidence was steeper under Strategy 2 than under Strategy 0 (Fig 1) and Strategy 2 was more cost-effective than Strategy 0.

In contrast to Strategies 0–2, Strategy 3 (which included both routine and catch-up HZ vaccination) showed a consistent decrease in annual HZ incidence and deaths over the entire 50-year time-horizon. Because this strategy incurred much higher vaccination costs due to HZ catch-up vaccination, the NMB of this strategy was lower than Strategy 2 under both payer and societal perspectives. Nevertheless, UVV with both routine and catch-up HZ vaccinations (Strategy 3) may be deemed a desirable public health policy option given the inclusion of catch-up HZ vaccination could successfully mitigate short-term increases in HZ outcomes due to varicella vaccination at a moderate cost to the society.

Our findings are consistent with other UVV modelling studies that account for exogenous boosting and dynamic demographics [42]. Because of the significant variation in local costs, methodology, and strategies considered, a detailed comparison of our findings with related UVV cost effectiveness analyses is beyond the scope of this manuscript. However, our findings were consistent with the findings of Anderson et al., who performed a systematic review of UVV cost effectiveness analyses and concluded that UVV was generally not expected to be cost-saving under the payer perspective but were potentially cost-saving under the societal perspective [21]. To address the impact of methodological differences in estimating the cost effectiveness of UVV, especially with respect to EB assumptions, a comparison study with a competing independently developed UVV DTM is underway and will be reported as future research.

Our findings are also consistent with other real-world evidence demonstrating significant decline in clinical and economic burden of varicella after implementation of UVV, including a cost-effectiveness analysis of UVV in Belgium [12]. Specifically, Bilcke and colleagues [12] found that a combination of UVV and adult zoster vaccination programs would be unlikely to result in a net QALY loss at any point during the time-horizon. However, because Bilcke and colleagues assumed that there was no zoster vaccination in their reference scenario, their incremental costs included the full cost of herpes zoster vaccination, and therefore, they found that UVV was unlikely to be cost effective over short time-horizons (i.e., under 30 years). Nevertheless, Bilcke and colleagues [12] identified a number of scenarios implementing a combination of UVV and adult zoster vaccination that would likely be cost-effective over a medium time-horizon (i.e., between 30 and 75 years). Additionally, countries such as the US, Germany, Spain, Italy, and Israel have reported significant reductions in both the clinical and economic burden of varicella over decades following implementation of UVV. Since 1995, in the United States the varicella vaccination program has led to the prevention of over 91 million varicella cases, 238 000 hospitalizations, and almost 2000 deaths, with a return on investment with net societal savings of more than $23 billion. Prior to the introduction of UVV, the annual incidence of HZ increased consistently since 1945 at an average rate of 2.5% per year [17]. Following the introduction of UVV, no significant acceleration in the rate of increase in annual HZ incidence has been observed over 25 years [17–19]. Indeed, annual HZ incidence plateaued sometime between 2012–2016 [18,19]. The magnitude of the secular trend in HZ (+2.5% per year) is sufficient to potentially obscure the modest increase in HZ incidence predicted by the model (approximately +2–3% versus baseline over 10 years). The increase in HZ incidence predicted by the model may be further obscured by the roll-out of UVV, which is instantaneous in the model versus gradual in the real world. Thus, while the data do not show any acceleration in the annual trend of increasing HZ incidence, these observations are not inconsistent with the model’s predictions. Moreover, given the observed plateau in HZ incidence, and given the prior secular trend, these data are consistent with the model’s prediction of declining HZ incidence in the medium- to long-term.

### Limitations

This model is subject to several limitations, some of which have been previously described [22]. First, this model implemented a temporary full immunity mechanism for exogenous boosting. However, multiple potential mechanisms for exogenous boosting are possible [14,43]. Since clinical and economic outcomes will be sensitive to the choice of exogenous boosting mechanism, this is a potentially significant source of model structural uncertainty. Unfortunately, our ability to apply model selection criteria to choose an appropriate exogenous boosting mechanism in an objective manner is limited by the lack of clinical and real-world data. Second, we assumed that there was no productivity loss for older adults aged ≥65 years and older. This is a standard economic assumption for older adults who are likely to be retired, however, a consequence of this assumption is that the benefits of vaccination strategies that include HZ vaccination may be underestimated under the societal perspective.

## Conclusion

Our model estimated a significant reduction in the clinical burden of varicella for various practical 2-dose UVV strategies. In Belgium, two-dose UVV without catch-up HZ vaccination and with early first-dose varicella catch-up was the most cost-effective UVV strategy at a WTP threshold of €40,000. In contrast, two-dose UVV with both routine and catch-up HZ vaccinations significantly reduced the burden of both varicella and HZ disease with a moderate increase in net monetary benefit versus the reference strategy. We therefore recommend that policymakers in Belgium consider the implementation of UVV programs as a cost-effective means to reduce the burden of varicella.

## Supporting information

S1 File Detailed explanation of demographic, epidemiological, and economic models, including model calibration and vaccine parameterization, sensitivity analyses, and additional results. (DOCX)
