# Supplementary material for: Modeling the clinical and economic impact of universal varicella vaccination in Belgium with dynamic population
Source: PLOS Glob Public Health. 2026 Jan 9;6(1):e0005636. doi: 10.1371/journal.pgph.0005636 (PMC12788688; doi:10.1371/journal.pgph.0005636)
Supplement: S1 File — (DOCX) [file pgph.0005636.s001.docx]

Modeling the Clinical and Economic Impact of Universal Varicella Vaccination in Belgium with Dynamic Population

Supplementary File 1

John C. Lang^1^*, Robert B. Nachbar^2^, Ilaria Xausa^2^, André Bento-Abreu^3^, Barbara Merckx^3^, Manjiri Pawaskar^4^

^1^ Health Economic and Decision Sciences (HEDS), Biostatistics and Research Decision Sciences (BARDS), Merck Canada Inc., Kirkland, QC, Canada

^2^ Wolfram Solutions, Wolfram Research, Inc., Champaign, IL, USA

^3^ Market Access, MSD Belgium BVBA, Brussels, VBR, Belgium

^4^ Value & Implementation Outcomes Research, Merck & Co., Inc., Rahway, NJ, USA

* Corresponding author

E-mail: [john.lang@merck.com](mailto:john.lang@merck.com)

Contents

[Table of Tables 4](#_Toc215995060)

[Table of Figures 5](#_Toc215995061)

[Section A Demographic Model 6](#_Toc215995062)

[A.1. Demographic Model Data 6](#_Toc215995063)

[A.1.1. Age Stratification 6](#_Toc215995064)

[A.1.2. Population 8](#_Toc215995065)

[A.1.3. Mortality 8](#_Toc215995066)

[A.1.4. Fertility 9](#_Toc215995067)

[A.1.5. Migration 10](#_Toc215995068)

[A.1.6. Contact Matrix 11](#_Toc215995069)

[A.2. Demographic Model Calibration 12](#_Toc215995070)

[Section B Epidemiological Model 14](#_Toc215995071)

[B.1. Summary 14](#_Toc215995072)

[B.2. Disease-Related Death Rates 16](#_Toc215995073)

[B.2.1. Varicella-Related Death Rate 16](#_Toc215995074)

[B.2.2. HZ Reactivation-Related Death Rate 16](#_Toc215995075)

[2.3 Ordinary Differential Equations 17](#_Toc215995076)

[Section C Calibration Data 23](#_Toc215995077)

[C.1. Varicella Seroprevalence 23](#_Toc215995078)

[C.2. Herpes Zoster Incidence 24](#_Toc215995079)

[Section D Economic Model 25](#_Toc215995080)

[D.1. Health Utility Outcomes 25](#_Toc215995081)

[D.2. Vaccination Costs 26](#_Toc215995082)

[D.3. Varicella Costs 26](#_Toc215995083)

[D.3.1. Varicella Direct Costs 26](#_Toc215995084)

[D.3.2. Indirect Varicella Costs 27](#_Toc215995085)

[D.4. Herpes Zoster Costs 27](#_Toc215995086)

[D.4.1. Herpes Zoster Direct Costs 27](#_Toc215995087)

[D.4.2. Herpes Zoster Indirect costs 30](#_Toc215995088)

[Section E Vaccine Parameterization 31](#_Toc215995089)

[E.1. Varicella Vaccine 31](#_Toc215995090)

[E.2. Herpes Zoster Vaccine 31](#_Toc215995091)

[Section F Vaccination Strategies 34](#_Toc215995092)

[Section G Sensitivity Analyses 36](#_Toc215995093)

[G.1. Scaling Factors 38](#_Toc215995094)

[G.1.1. Costs 38](#_Toc215995095)

[G.1.2. Quality-adjusted life-years (QALY) 38](#_Toc215995096)

[Section H Additional Results 39](#_Toc215995097)

[H.1. Model Calibration 39](#_Toc215995098)

[H.1.1. Demographic Model 39](#_Toc215995099)

[H.1.2. Epidemiological Model 41](#_Toc215995100)

[H.2. Clinical and Economic Outcomes 43](#_Toc215995101)

[H.3. Sensitivity analyses 44](#_Toc215995102)

[References 50](#_Toc215995103)

# Table of Tables

[Table A: Age stratification for the Belgium VZV DTM adaptation 6](#_Toc215995104)

[Table B: Population by age group and year (1992-2075) for Belgium* 8](#_Toc215995105)

[Table C: Annual probability of dying by age group and year (1992-2017 for Belgium* 8](#_Toc215995106)

[Table D: Fertility by age group and year (1992-2075) for Belgium* 9](#_Toc215995107)

[Table E: Per capita migration by age group for Belgium 10](#_Toc215995108)

[Table F: Summary of epidemiological parameters 14](#_Toc215995109)

[Table G: Varicella case fatality by age 16](#_Toc215995110)

[Table H: Herpes zoster case fatality by age 16](#_Toc215995111)

[Table I: Definition of model variables 18](#_Toc215995112)

[Table J: Varicella seroprevalence in Belgium 23](#_Toc215995113)

[Table K: Herpes zoster general practice consultation incidence (2006-2008; 2012-2018) 24](#_Toc215995114)

[Table L: Healthy QALY values 25](#_Toc215995115)

[Table M: QALY weights and distribution of herpes zoster cases by pain severity 25](#_Toc215995116)

[Table N: Proportion of herpes zoster cases that develop PHN 26](#_Toc215995117)

[Table O: Pediatric vaccine costs (2023 Euros) 26](#_Toc215995118)

[Table P: Fraction of varicella infections seeking GP care 27](#_Toc215995119)

[Table Q: Fraction of varicella infections that result in hospitalization 27](#_Toc215995120)

[Table R: Herpes zoster outpatient and inpatient resource use 28](#_Toc215995121)

[Table S: Joint distribution for herpes zoster hospitalization, PHN status, and pain severity 29](#_Toc215995122)

[Table T: Herpes zoster costs by hospitalization status, PHN status, and pain severity (2023 Euros) 29](#_Toc215995123)

[Table U: Summary of herpes zoster GP and hospitalization costs by age (2023 Euros) 30](#_Toc215995124)

[Table V: Herpes zoster vaccine efficacy 31](#_Toc215995125)

[Table W: Vaccination strategies 35](#_Toc215995126)

[Table X: Parameter distributions for sensitivity analyses 36](#_Toc215995127)

[Table Y: Cumulative clinical outcomes (2023-2073) 43](#_Toc215995128)

[Table Z: Marginal cost and QALY outcomes (2023-2073) 43](#_Toc215995129)

# Table of Figures

[Fig A: Demographic model calibration results 12](#_Toc215995130)

[Fig B: Demographic model projection (2023-2075) 13](#_Toc215995131)

[Fig C: Disease and vaccination structure for dynamic transmission model 17](#_Toc215995132)

[Fig D: Bootstrap estimates of herpes zoster vaccine waning parameter ($\omega z$) 32](#_Toc215995133)

[Fig E: Herpes zoster vaccine efficacy 33](#_Toc215995134)

[Fig F: Demographic structure (model output versus calibration data, 1992-2023) 39](#_Toc215995135)

[Fig G: Demographic structure (model projection, 2023-2075) 40](#_Toc215995136)

[Fig H: Pre-UVV varicella seroprevalence (2017) 41](#_Toc215995137)

[Fig I: Herpes zoster incidence (2006-2008 and 2012-2018) 42](#_Toc215995138)

[Fig J: Tornado diagram for Strategy 1 incremental cost-effectiveness deterministic sensitivity analysis (DSA) results 44](#_Toc215995139)

[Fig K: Tornado diagram for Strategy 2 incremental cost-effectiveness deterministic sensitivity analysis (DSA) results 45](#_Toc215995140)

[Fig L: Tornado diagram for Strategy 3 incremental cost-effectiveness deterministic sensitivity (DSA) results 46](#_Toc215995141)

[Fig M: Marginal cost versus marginal QALY scatter plot for Strategy 1 probabilistic sensitivity analysis (PSA) results 47](#_Toc215995142)

[Fig N: Marginal cost versus marginal QALY scatter plot for Strategy 2 probabilistic sensitivity analysis (PSA) results 48](#_Toc215995143)

[Fig O: Marginal cost versus marginal QALY scatter plot for Strategy 3 probabilistic sensitivity analysis (PSA) results 49](#_Toc215995144)

# Demographic Model

## Demographic Model Data

### Age Stratification

Table A: Age stratification for the Belgium VZV DTM adaptation

| **Age group**  **(j)** | **Minimum Age**  **(years)** | **Maximum Age**  **(years)** | **Description** |
| --- | --- | --- | --- |
| 1 | 0.00 | 0.08 | 0-1 months |
| 2 | 0.08 | 0.25 | 1-3 months |
| 3 | 0.25 | 0.50 | 3-6 months |
| 4 | 0.50 | 0.75 | 6-9 months |
| 5 | 0.75 | 0.92 | 9-11 months |
| 6 | 0.92 | 1.00 | 11-12 months |
| 7 | 1.00 | 1.08 | 12-13 months |
| 8 | 1.08 | 1.17 | 13-14 months |
| 9 | 1.17 | 1.25 | 14-15 months |
| 10 | 1.25 | 1.33 | 15-16 months |
| 11 | 1.33 | 1.42 | 16-17 months |
| 12 | 1.42 | 1.50 | 17-18 months |
| 13 | 1.50 | 1.58 | 18-19 months |
| 14 | 1.58 | 1.67 | 19-20 months |
| 15 | 1.67 | 1.75 | 20-21 months |
| 16 | 1.75 | 1.83 | 21-22 months |
| 17 | 1.83 | 1.92 | 22-23 months |
| 18 | 1.92 | 2.00 | 23-24 months |
| 19 | 2.00 | 3.00 | 24-36 months |
| 20 | 3.00 | 3.33 | 36-40 months |
| 21 | 3.33 | 4.00 | 40-48 months |
| 22 | 4.00 | 5.00 | 48-60 months |
| 23 | 5.00 | 5.50 | 60-66 months |
| 24 | 5.50 | 6 | 66-72 months |
| 25 | 6 | 7 | 6-7 years |
| 26 | 7 | 8 | 7-8 years |
| 27 | 8 | 9 | 8-9 years |
| 28 | 9 | 10 | 9-10 years |
| 29 | 10 | 11 | 10-11 years |
| 30 | 11 | 12 | 11-12 years |
| 31 | 12 | 13 | 12-13 years |
| 32 | 13 | 14 | 13-14 years |
| 33 | 14 | 15 | 14-15 years |
| 34 | 15 | 16 | 15-16 years |
| 35 | 16 | 17 | 16-17 years |
| 36 | 17 | 18 | 17-18 years |
| 37 | 18 | 19 | 18-19 years |
| 38 | 19 | 20 | 19-20 years |
| 39 | 20 | 25 | 20-25 years |
| 40 | 25 | 30 | 25-30 years |
| 41 | 30 | 35 | 30-35 years |
| 42 | 35 | 40 | 35-40 years |
| 43 | 40 | 45 | 40-45 years |
| 44 | 45 | 50 | 45-50 years |
| 45 | 50 | 55 | 50-55 years |
| 46 | 55 | 60 | 55-60 years |
| 47 | 60 | 65 | 60-65 years |
| 48 | 65 | 70 | 65-70 years |
| 49 | 70 | 75 | 70-75 years |
| 50 | 75 | 80 | 75-80 years |
| 51 | 80 | 85 | 80-85 years |
| 52 | 85 | 90 | 85-90 years |
| 53 | 90 | Infinity | 90+ years |

### Population

Population estimates were available for yearly cohorts from 1992-2071 [1], see Table B (note: this table is embedded as a CSV file). For years 2072-2075 the population was estimated by extrapolating from a linear regression of the logarithm of population versus the year for years 2062-2071.

Table B: Population by age group and year (1992-2075) for Belgium*

* Note: This table is embedded as a CSV file.

### Mortality

Mortality estimates were available for yearly cohorts from 1992-2070 [2], see Table C (note: this table is embedded as a CSV file). For years 2071-2075 the mortality was estimated by extrapolating from a linear regression of the logarithm of mortality versus the year for years 2061-2070.

Table C: Annual probability of dying by age group and year (1992-2017 for Belgium*

* Note: This table is embedded as a CSV file.

### Fertility

Fertility estimates were available for yearly cohorts from 1992-2075 [3], see Table D (note: this table is embedded as a CSV file). Estimates of total annual births were available from 1992-2070 [4]. For years 2071-2075 the total annual births were estimated by extrapolating from a linear regression of the logarithm of mortality versus the year for years 2061-2070.

Table D: Fertility by age group and year (1992-2075) for Belgium*

* Note: This table is embedded as a CSV file.

### Migration

Migration was assumed to be constant throughout the model simulation. Migration rates by age were calculated using the *mig_un_fam* function of the *DemoTools* R package [5]. The *mig_un_fam* function implemented the algorithm documented by Rogers and Castro (1981) [6] and required three arguments: the total number of migrants for the year 2011 [4], the predominant type of immigration (assumed to be “Family”, i.e., family reunion), and the final age strata (all ages 100+ were grouped together)**.** Finally, per capita migration was calculated using population estimates from 2011 [1].

Table E: Per capita migration by age group for Belgium

| **Age**  **(years)** | **Migration**  **(per capita)** | **Age**  **(years)** | **Migration**  **(per capita)** |
| --- | --- | --- | --- |
| 0 | 0.00617 | 46 | 0.00248 |
| 1 | 0.00527 | 47 | 0.00232 |
| 2 | 0.00445 | 48 | 0.00217 |
| 3 | 0.00385 | 49 | 0.00200 |
| 4 | 0.00329 | 50 | 0.00185 |
| 5 | 0.00286 | 51 | 0.00170 |
| 6 | 0.00247 | 52 | 0.00160 |
| 7 | 0.00216 | 53 | 0.00150 |
| 8 | 0.00186 | 54 | 0.00141 |
| 9 | 0.00158 | 55 | 0.00131 |
| 10 | 0.00141 | 56 | 0.00123 |
| 11 | 0.00144 | 57 | 0.00116 |
| 12 | 0.00171 | 58 | 0.00108 |
| 13 | 0.00235 | 59 | 0.00104 |
| 14 | 0.00345 | 60 | 0.00096 |
| 15 | 0.00503 | 61 | 0.00089 |
| 16 | 0.00683 | 62 | 0.00082 |
| 17 | 0.00850 | 63 | 0.00077 |
| 18 | 0.00998 | 64 | 0.00071 |
| 19 | 0.01139 | 65 | 0.00078 |
| 20 | 0.01271 | 66 | 0.00074 |
| 21 | 0.01384 | 67 | 0.00072 |
| 22 | 0.01444 | 68 | 0.00077 |
| 23 | 0.01487 | 69 | 0.00079 |
| 24 | 0.01478 | 70 | 0.00066 |
| 25 | 0.01480 | 71 | 0.00057 |
| 26 | 0.01410 | 72 | 0.00053 |
| 27 | 0.01344 | 73 | 0.00052 |
| 28 | 0.01244 | 74 | 0.00051 |
| 29 | 0.01156 | 75 | 0.00049 |
| 30 | 0.01072 | 76 | 0.00046 |
| 31 | 0.01011 | 77 | 0.00045 |
| 32 | 0.00951 | 78 | 0.00042 |
| 33 | 0.00892 | 79 | 0.00041 |
| 34 | 0.00832 | 80 | 0.00040 |
| 35 | 0.00779 | 81 | 0.00043 |
| 36 | 0.00697 | 82 | 0.00044 |
| 37 | 0.00623 | 83 | 0.00046 |
| 38 | 0.00554 | 84 | 0.00047 |
| 39 | 0.00497 | 85 | 0.00049 |
| 40 | 0.00451 | 86 | 0.00053 |
| 41 | 0.00418 | 87 | 0.00057 |
| 42 | 0.00384 | 88 | 0.00065 |
| 43 | 0.00349 | 89 | 0.00073 |
| 44 | 0.00312 | 90 | 0.00234 |
| 45 | 0.00279 |  |  |

### Contact Matrix

We used the Belgian contact matrix estimated by Prem and colleagues [7].

## Demographic Model Calibration


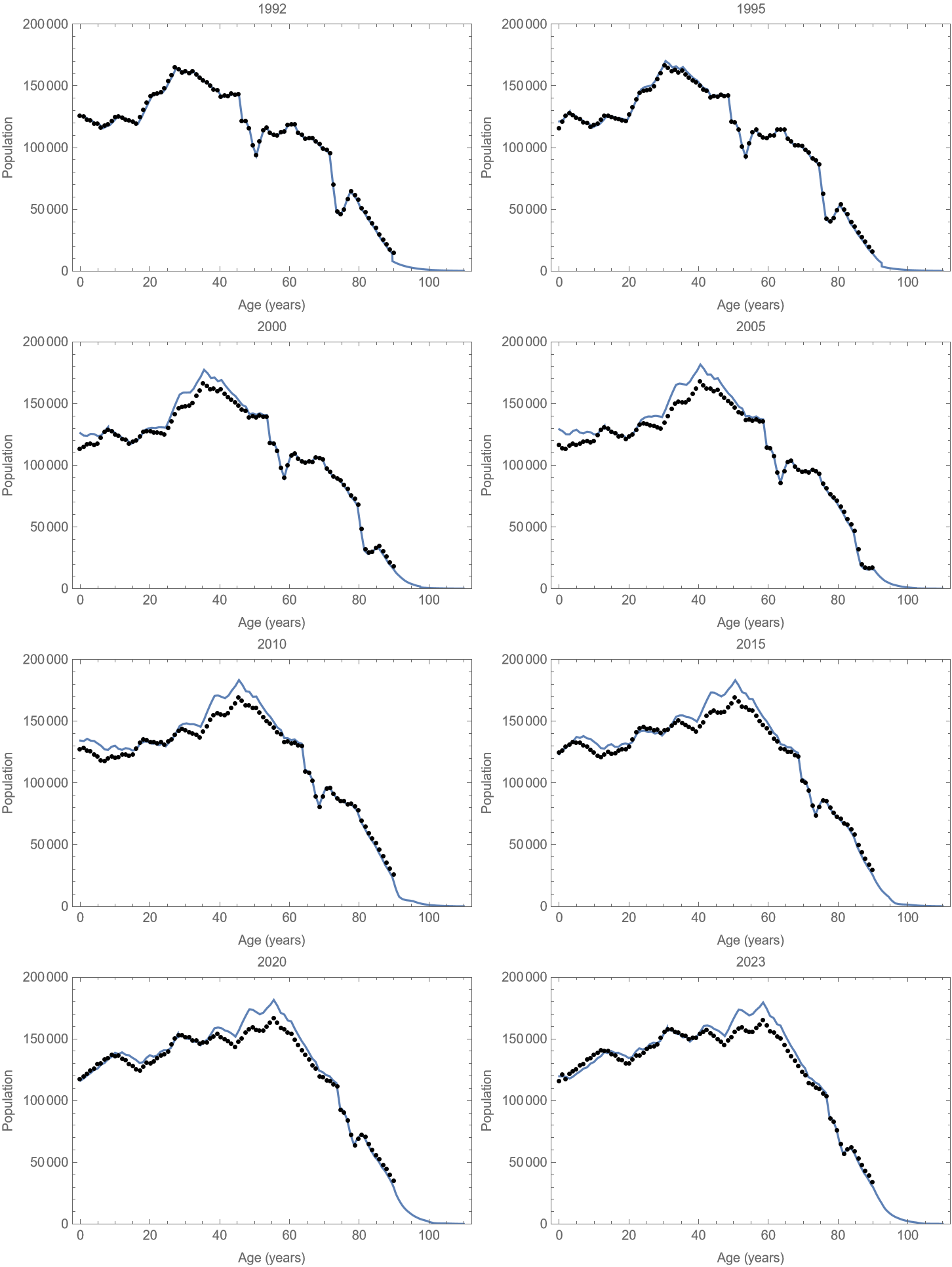


Fig A: Demographic model calibration results

***(Dots) Population data. (Line) Model output.***


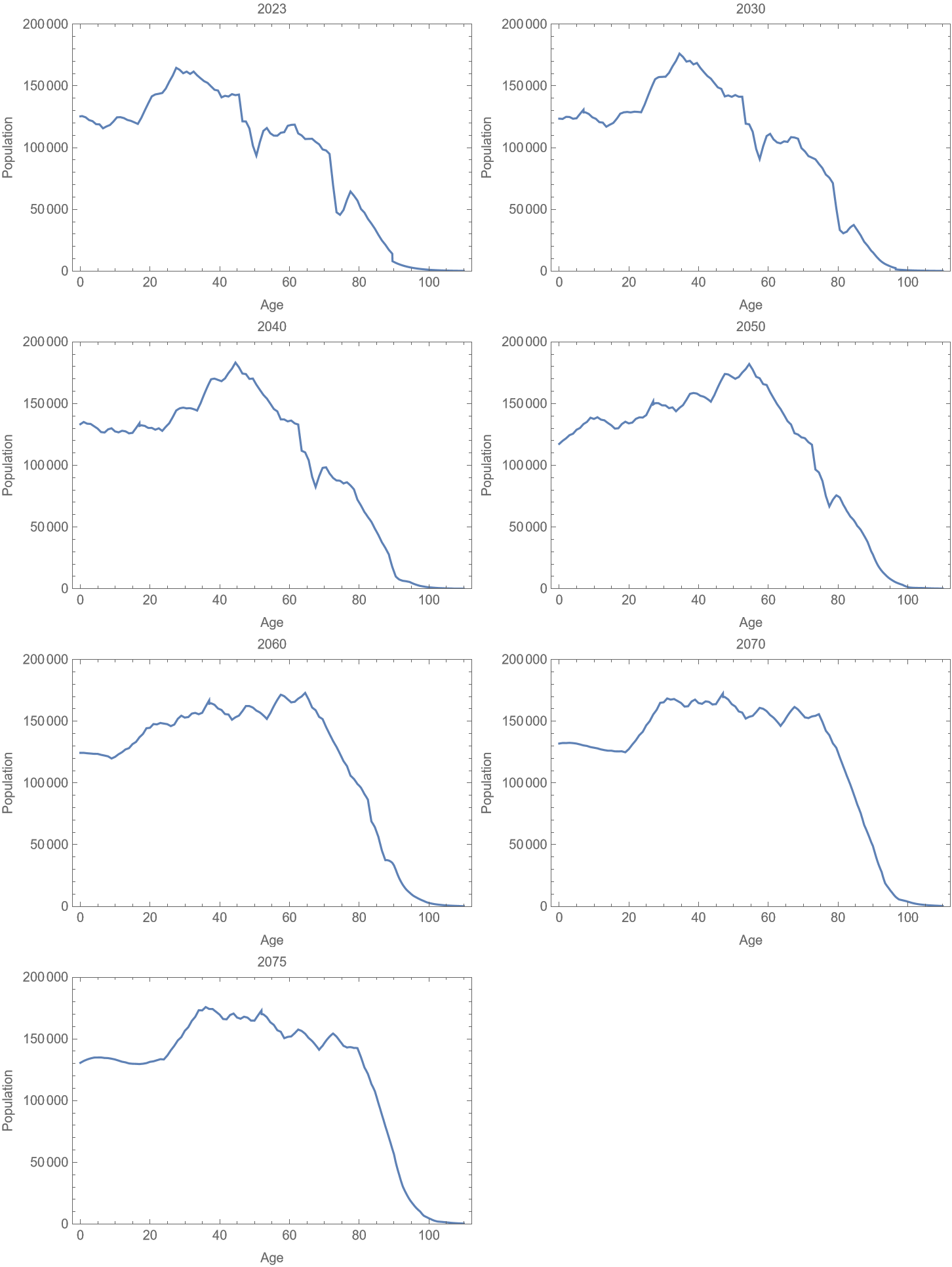


Fig B: Demographic model projection (2023-2075)

# Epidemiological Model

## Summary

The following table summarizes the differences in epidemiological parameters used in this model versus those reported by Sharomi and colleagues (i.e., Table 1 of [8]).

Table F: Summary of epidemiological parameters

| **Symbol** | **Parameter Description** | **Value** | **Source** |  |
| --- | --- | --- | --- | --- |
| ***Natural Varicella Infection*** | | | | |
| $\frac{1}{\omega^{m}}$ | Duration of maternal immunity | 6 months | [9, 10]^A^ |  |
| $\frac{1}{\epsilon^{n}}$ | Duration of latent period | 14 days | [9, 11, 12]^A^ |  |
| $\frac{1}{\gamma^{n}}$ | Duration of infectious period | 7 days | [9, 13]^A^ |  |
| $\frac{1}{\delta^{n}}$ | Duration of HZ immunity | 81.3 years | [14], supplement^A^ |  |
| ***Breakthrough Varicella Infection*** | | | | |
| $\rho^{v}$ | Relative infectivity of breakthrough varicella | 50% | [15]^A^ |  |
| $\frac{1}{\epsilon^{vb}}$ | Duration of latent period | 14 days | [9, 11]^A^ |  |
| $\frac{1}{\gamma^{vb}}$ | Duration of infectious period | 4.5 days | [9, 13]^A^ |  |
| $\frac{1}{\delta^{vb}}$ | Duration of HZ immunity | 81.3 years | [14], supplement^A^ |  |
| ***Varicella Susceptibility*** | | | | |
| *δ* | Ramp parameter | 0 | Assumption |  |
| *rr1* | Relative risk (<5 years old) | 0.0528 | Calibrated |  |
| *rr2* | Relative risk (5-10 years old) | 0.1323 | Calibrated |  |
| *rr3* | Relative risk (10-20 years old) | 0.0093 | Calibrated |  |
| *rr4* | Relative risk (≥20 years old) | 0.0580 | Calibrated |  |
| ***HZ Reactivation*** | | | | |
| $\rho^{z}$ | Relative infectivity of HZ | 7% |  |  |
| *ω* | HZ Reactivation parameter | 0.3365 | Calibrated |  |
| *φ* | HZ Reactivation parameter | 0.1171 | Calibrated |  |
| *η* | HZ Reactivation parameter | 3.0686 | Calibrated |  |
| *π* | HZ Reactivation parameter | 0.0142 | Calibrated |  |
| $\chi$ | Reactivation rate factor on vaccine arms | 1/6 | [16]^A^ |  |
| $\frac{1}{\eta^{n}}$ | Duration of infectious period for HZ outbreak (natural varicella) | 28 days | [9, 17]^A^ |  |
| $\frac{1}{\eta^{vb}}$ | Duration of infectious period for HZ outbreak (breakthrough varicella) | 28 days | [9, 17]^A^ |  |
| $\frac{1}{\eta^{vv}}$ | Duration of infectious period for HZ outbreak (following vaccination) | 28 days | [9, 17]^A^ |  |
| $\frac{1}{\delta^{vv}}$ | Duration of exogenous boosting HZ immunity following vaccination | 81.3 years | [14], supplement^A^ |  |
| ***Contacts Leading to Exogenous Boosting*** | | | | |
| $\zeta^{n}$ | After natural varicella | 33.45% | [14], supplement^A^ |  |
| $\zeta^{vb}$ | After breakthrough varicella | 33.45% | [14], supplement^A^ |  |
| $\zeta^{vv}$ | After varicella vaccination | 33.45% | [14], supplement^A^ |  |
| ***Disease-Related Death Rates, by year of age group*** | | | | |
| *d_j_^v^* | Natural varicella death rate | Various | See Section 2.2.1 |  |
| *d_j_^vb^* | Breakthrough varicella death rate | 0/year | Assumption |  |
| *d_j_^z^* | HZ reactivation death rate | Various | See Section 2.2.2 |  |

^A^ Unchanged from Sharomi and colleagues (i.e., Table 1 of [8]).

## Disease-Related Death Rates

### Varicella-Related Death Rate

The varicella-related death rate for age strata *j*, i.e., *d^v^_j_*, was computed using the formula

$$\left( case fatality rate \right)_{j}=\frac{d_{j}^{v}}{\gamma^{n}+d_{j}^{v}+\mu_{j}}\Leftrightarrow d_{j}^{v}=\frac{\left( case fatality rate \right)_{j}}{1-\left( case fatality rate \right)_{j}}\times(\gamma^{n}+\mu_{j}) ,$$

where *γ^n^* is the recovery rate and *μ_j_* is the background mortality rate for age strata *j* (for year 1997). The case fatality rates for varicella infections were taken from Riera-Montes and colleagues [18] (see TableG).

Table G: Varicella case fatality by age

| **Age (years)** | **Case Fatality Rate (per 100,000)** |
| --- | --- |
| 0-5 | 0.53 |
| 5-10 | 0.28 |
| 10-15 | 1.14 |
| 15-20 | 0.86 |
| 20-40 | 4.90 |
| ≥40 | 31.90 |

### HZ Reactivation-Related Death Rate

The HZ reactivation-related death rate for age strata *j*, i.e., *d^z^_j_*, was computed using the formula

$$\left( case fatality rate \right)_{j}=\frac{d_{j}^{z}}{\eta^{n}+d_{j}^{z}+\mu_{j}}\Leftrightarrow d_{j}^{z}=\frac{\left( case fatality rate \right)_{j}}{1-\left( case fatality rate \right)_{j}}\times(\eta^{n}+\mu_{j}) ,$$

where *η^n^* is the recovery rate for reactivated HZ and *μ_j_* is the background mortality rate for age strata *j* (for year 1997). The case fatality rates for varicella infections were taken from Pieters and colleagues [19] (see Table H).

Table H: Herpes zoster case fatality by age

| **Age (years)** | **Case Fatality Rate (per 100,000)** |
| --- | --- |
| 0-60 | 0 |
| 60-70 | 2.15 |
| 70-75 | 1.79 |
| 75-80 | 47.43 |
| 80-90 | 49.55 |
| ≥90 | 90.34 |

## 2.3 Ordinary Differential Equations

For additional details see Sharomi, et al., (2022) [8].


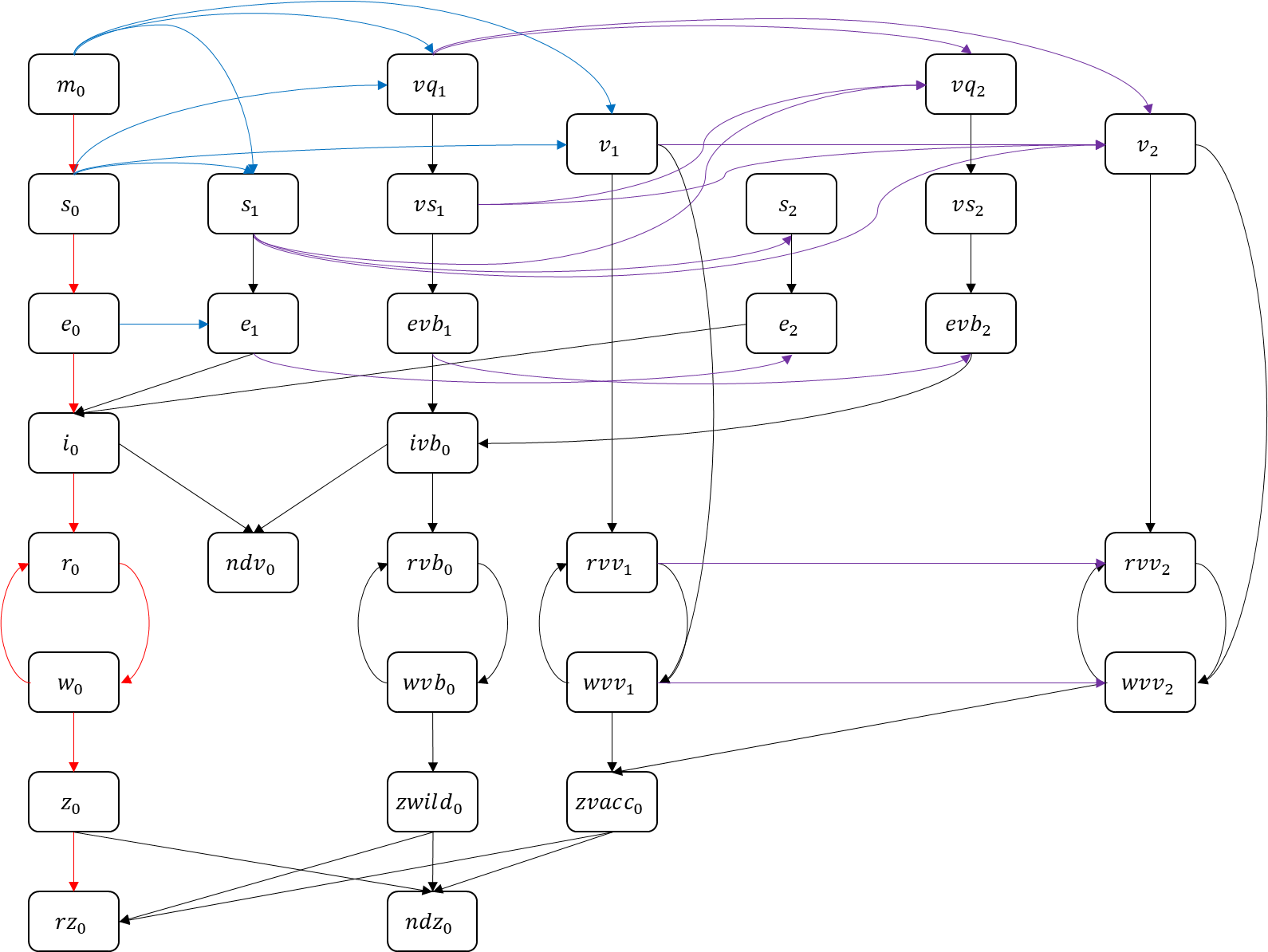


Fig C: Disease and vaccination structure for dynamic transmission model

**Blue lines represent vaccination with UVV first dose, purple lines represent vaccination with second dose, red lines represent varicella natural history, and black lines represent all other transitions in the figure.**

Table I: Definition of model variables

| **Variable** | **Description (variables represent proportions)** |
| --- | --- |
| **Unvaccinated Compartments** | |
| $m_{j}$ | passively immune |
| $s_{j}$ | susceptible to varicella infection |
| $e_{j}$ | latent varicella |
| $i_{j}$ | infectious varicella |
| $r_{j}$ | recovered from varicella with high HZ immunity |
| $w_{j}$ | low HZ immunity due to waning effects |
| **Varicella Vaccination** | |
| $v_{j,l}$ | long-lasting immunity following $l-$dose vaccination |
| ${vq}_{j,l}$ | temporary immunity following $l-$dose vaccination |
| ${vs}_{j,l}$ | susceptible to varicella following $l-$dose vaccine waning |
| $s_{j,l}$ | susceptible to varicella following $l-$dose vaccine failure |
| $e_{j,l}$ | latent varicella following $l-$dose vaccine failure |
| ${rvv}_{j,l}$ | High HZ immunity following $l-$dose vaccination |
| ${wvv}_{j,l}$ | low HZ immunity following $l-$dose vaccine waning |
| **Breakthrough Varicella** | |
| ${evb}_{j,l}$ | latent varicella following $l-$dose vaccine waning |
| ${ivb}_{j}$ | infectious varicella |
| ${rvb}_{j}$ | recovered from varicella with high HZ immunity |
| ${wvb}_{j}$ | low HZ immunity due to waning |
| **HZ Reactivation** | |
| $z_{j}$ | infectious with wild type HZ |
| ${zvacc}_{j}$ | infectious with wild type HZ post varicella vaccination |
| ${zwild}_{j}$ | infectious with wild type HZ post breakthrough varicella |
| ${rz}_{j}$ | recovered from HZ with high HZ immunity |
| **Death** | |
| ${ndv}_{j}$ | Death from varicella |
| ${ndz}_{j}$ | Death from HZ |

$$\frac{{dm}_{j}}{dt}= B^{m}\left( t \right)\delta_{1,j}+d_{j-1}\left( t \right)\left( {1-\delta}_{1,j} \right)\left( 1-\theta_{j}^{p}\left( t \right)-\theta_{j}^{c}\left( t \right)-\theta_{j}^{s}\left( t \right) \right)m_{j-1}\left( t \right) -\left( d_{j}\left( t \right)+\omega^{m}+\mu_{j}\left( t \right) \right)m_{j}\left( t \right).$$

$$\frac{{ds}_{j}}{dt}= B^{s}\left( t \right)\delta_{1,j}+ \omega^{m}m_{j}\left( t \right)+d_{j-1}\left( t \right)\left( 1-\delta_{1,j} \right)\left( 1-\theta_{j}^{p}\left( t \right)-\theta_{j}^{c}\left( t \right)-\theta_{j}^{s}\left( t \right) \right)s_{j-1}\left( t \right) -\left( d_{j}\left( t \right)+\mu_{j}\left( t \right)+\lambda_{j}\left( t \right) \right)s_{j}\left( t \right).$$

$$\frac{{de}_{j}}{dt}= \lambda_{j}\left( t \right)s_{j}\left( t \right)+d_{j-1}\left( t \right)\left( {1-\delta}_{1,j} \right)\left( 1-\theta_{j}^{p}\left( t \right)-\theta_{j}^{c}\left( t \right)-\theta_{j}^{s}\left( t \right) \right)e_{j-1}\left( t \right) -\left( d_{j}\left( t \right)+\mu_{j}\left( t \right)+\epsilon^{n} \right)e_{j}\left( t \right).$$

$$\frac{{di}_{j}}{dt}= d_{j-1}\left( t \right)\left( {1-\delta}_{1,j} \right)i_{j-1}\left( t \right)+\epsilon^{n}\left( e_{j}\left( t \right)+ e_{j,1}\left( t \right)+e_{j,2}\left( t \right) \right)- \left( d_{j}\left( t \right)+\mu_{j}\left( t \right)+\gamma^{n}+d_{j}^{v} \right)i_{j}\left( t \right).$$

$$\frac{{dr}_{j}}{dt}= d_{j-1}\left( t \right)\left( {1-\delta}_{1,j} \right)r_{j-1}\left( t \right) +\left( {\xi^{n}}_{j}+{\zeta^{n}}_{j}\lambda_{j} \right)\left( t \right)w_{j}\left( t \right)+ \gamma^{n}i_{j}\left( t \right)-\left( d_{j}\left( t \right)+\mu_{j}\left( t \right)+\delta^{n}+bxz \sigma_{j} \right)r_{j}\left( t \right).$$

$$\frac{{dw}_{j}}{dt}=d_{j-1}\left( t \right)\left( {1-\delta}_{1,j} \right)\left( 1-\theta_{j}^{z}\left( t \right) \right)w_{j-1}\left( t \right)+\delta^{n}r_{j}\left( t \right)-\left( d_{j}\left( t \right)+\mu_{j}\left( t \right)+\sigma_{j}+{\xi^{n}}_{j}+{\zeta^{n}}_{j}\lambda_{j}\left( t \right) \right)w_{j}\left( t \right).$$

$$\frac{{dv}_{j,1}}{dt}=d_{j-1}\left( t \right)\left( {1-\delta}_{1,j} \right)v_{j-1,1}\left( t \right)+d_{j-1}\left( t \right)\left( {1-\delta}_{1,j} \right)\left[ \left( \theta_{j}^{p}\left( t \right)+\theta_{j}^{c}\left( t \right)+\theta_{j}^{s}\left( t \right) \right)PT_{1}\left( m_{j-1}\left( t \right)+s_{j-1}\left( t \right) \right)-\left( \theta_{j}^{b}\left( t \right)+\theta_{j}^{d}\left( t \right) \right)v_{j-1,1}\left( t \right) \right]-\left( d_{j}\left( t \right)+\mu_{j}\left( t \right)+\pi_{1}+k_{1}\lambda_{j}\left( t \right) \right)v_{j,1}\left( t \right).$$

$$\frac{{dv}_{j,2}}{dt}=d_{j-1}\left( t \right)\left( {1-\delta}_{1,j} \right)v_{j-1,2}\left( t \right)++d_{j-1}\left( t \right)\left( {1-\delta}_{1,j} \right)\left( \theta_{j}^{b}\left( t \right)+\theta_{j}^{d}\left( t \right) \right)\left[ v_{j-1,1}\left( t \right)+PT_{1}s_{j-1,1}\left( t \right)+T_{2}\left( {vq}_{j-1,1}\left( t \right)+{vs}_{j-1,1}\left( t \right) \right) \right]-\left( d_{j}\left( t \right)+\mu_{j}\left( t \right)+\pi_{2}+k_{2}\lambda_{j}\left( t \right) \right)v_{j,2}\left( t \right).$$

$$\frac{{dvq}_{j,1}}{dt}=d_{j-1}\left( t \right)\left( {1-\delta}_{1,j} \right){vq}_{j-1,1}\left( t \right)+d_{j-1}\left( t \right)\left( {1-\delta}_{1,j} \right)\left[ P\left( 1-T_{1} \right)\left( \theta_{j}^{p}\left( t \right)+\theta_{j}^{c}\left( t \right)+\theta_{j}^{s}\left( t \right) \right)\left( m_{j-1}\left( t \right)+s_{j-1}\left( t \right) \right)-\left( \theta_{j}^{b}\left( t \right)+\theta_{j}^{d}\left( t \right)+\theta_{j}^{z}\left( t \right) \right){vq}_{j-1,1}\left( t \right) \right]-\left( d_{j}\left( t \right)+\mu_{j}\left( t \right)+\sigma^{v} \right){vq}_{j,1}\left( t \right).$$

$$\frac{{dvq}_{j,2}}{dt}=d_{j-1}\left( t \right)\left( {1-\delta}_{1,j} \right)\left( 1-\theta_{j}^{z}\left( t \right) \right){vq}_{j-1,2}\left( t \right)+d_{j-1}\left( t \right)\left( {1-\delta}_{1,j} \right)\left( \theta_{j}^{b}\left( t \right)+\theta_{j}^{d}\left( t \right) \right)\left[ P\left( 1-T_{1} \right)s_{j-1,1}\left( t \right)+\left( 1-T_{2} \right)\left( {vq}_{j-1,1}\left( t \right)+{vs}_{j-1,1}\left( t \right) \right) \right]-\left( d_{j}\left( t \right)+\mu_{j}\left( t \right)+\sigma^{v} \right){vq}_{j,2}\left( t \right).$$

$$\frac{{dvs}_{j,1}}{dt}= \sigma^{v}{vq}_{j,1}\left( t \right)+d_{j-1}\left( t \right)\left( {1-\delta}_{1,j} \right)\left( 1-\theta_{j}^{b}\left( t \right)-\theta_{j}^{d}\left( t \right)-\theta_{j}^{z}\left( t \right) \right){vs}_{j-1,1}\left( t \right)-\left( d_{j}\left( t \right)+\mu_{j}\left( t \right)+\lambda_{j}\left( t \right) \right){vs}_{j,1}\left( t \right).$$

$$\frac{{dvs}_{j,2}}{dt}= \sigma^{v}{vq}_{j,2}\left( t \right)+d_{j-1}\left( t \right)\left( {1-\delta}_{1,j} \right)\left( 1-\theta_{j}^{z}\left( t \right) \right){vs}_{j-1,2}\left( t \right)-\left( d_{j}\left( t \right)+\mu_{j}\left( t \right)+\lambda_{j}\left( t \right) \right){vs}_{j,2}\left( t \right).$$

$$\frac{{dvzv}_{j}}{dt}= d_{j-1}\left( t \right)\left( {1-\delta}_{1,j} \right){vzv}_{j-1}\left( t \right)-Tzd_{j-1}\left( t \right)\left( {1-\delta}_{1,j} \right)\theta_{j}^{z}\left( t \right)\left( {vq}_{j-1,1}\left( t \right)+{vq}_{j-1,2}\left( t \right)+{vs}_{j-1,1}\left( t \right)+{vs}_{j-1,2}\left( t \right)+{wvv}_{j-1,1}\left( t \right)+{wvv}_{j-1,2}\left( t \right) \right)-\left( \omega z+d_{j}\left( t \right)+\mu_{j}\left( t \right)+\mathrm{bz}\chi\sigma_{j}\left( t \right) \right){vzv}_{j}\left( t \right),$$

$$\frac{{dvzw}_{j}}{dt}= d_{j-1}\left( t \right)\left( {1-\delta}_{1,j} \right)\left( {vzw}_{j-1}\left( t \right)+Tz \theta_{j}^{z}\left( t \right)w_{j-1}\left( t \right) \right)-\left( \omega^{z}+d_{j}\left( t \right)+\mu_{j}\left( t \right)+\mathrm{bz}\sigma_{j}\left( t \right) \right){vzw}_{j}\left( t \right),$$

$$\frac{{dvzb}_{j}}{dt}= d_{j-1}\left( t \right)\left( {1-\delta}_{1,j} \right)\left( {vzb}_{j-1}\left( t \right)+Tz \theta_{j}^{z}\left( t \right){wvb}_{j-1}\left( t \right) \right)-\left( \omega^{z}+d_{j}\left( t \right)+\mu_{j}\left( t \right)+\mathrm{bz}\chi\sigma_{j}\left( t \right) \right){vzb}_{j}\left( t \right),$$

$$\frac{{ds}_{j,1}}{dt}= d_{j-1}\left( t \right)\left( {1-\delta}_{1,j} \right)s_{j-1,1}\left( t \right)+d_{j-1}\left( t \right)\left( {1-\delta}_{1,j} \right)\left[ \left( 1-P \right)\left( \theta_{j}^{p}\left( t \right)+\theta_{j}^{c}\left( t \right)+\theta_{j}^{s}\left( t \right) \right)\left( m_{j-1}\left( t \right)+s_{j-1}\left( t \right) \right)-\left( \theta_{j}^{b}\left( t \right)+\theta_{j}^{d}\left( t \right) \right)s_{j-1,1}\left( t \right) \right]-\left( d_{j}\left( t \right)+\mu_{j}\left( t \right)+\lambda_{j}\left( t \right) \right)s_{j,1}\left( t \right).$$

$$\frac{{ds}_{j,2}}{dt}= d_{j-1}\left( t \right)\left( {1-\delta}_{1,j} \right)s_{j-1,2}\left( t \right)+d_{j-1}\left( t \right)\left( {1-\delta}_{1,j} \right)\left( 1-P \right)\left( \theta_{j}^{b}\left( t \right)+\theta_{j}^{d}\left( t \right) \right)s_{j-1,1}\left( t \right)-\left( d_{j}\left( t \right)+\mu_{j}\left( t \right)+\lambda_{j}\left( t \right) \right)s_{j,2}\left( t \right).$$

$$\frac{{de}_{j,1}}{dt}=d_{j-1}\left( t \right)\left( {1-\delta}_{1,j} \right)e_{j-1,1}\left( t \right)+d_{j-1}\left( t \right)\left( {1-\delta}_{1,j} \right)\left[ \left( \theta_{j}^{p}\left( t \right)+\theta_{j}^{c}\left( t \right)+\theta_{j}^{s}\left( t \right) \right)e_{j-1}\left( t \right)-\left( \theta_{j}^{b}\left( t \right)+\theta_{j}^{d}\left( t \right) \right)e_{j-1,1}\left( t \right) \right]+\lambda_{j}\left( t \right)s_{j,1}\left( t \right)- \left( d_{j}\left( t \right)+\mu_{j}\left( t \right)+\epsilon^{n} \right)e_{j,1}\left( t \right).$$

$$\frac{{de}_{j,2}}{dt}=d_{j-1}\left( t \right)\left( {1-\delta}_{1,j} \right)e_{j-1,2}\left( t \right)+d_{j-1}\left( t \right)\left( {1-\delta}_{1,j} \right)\left( \theta_{j}^{b}\left( t \right)+\theta_{j}^{d}\left( t \right) \right)e_{j-1,1}\left( t \right)+\lambda_{j}\left( t \right)s_{j,2}\left( t \right)- \left( d_{j}\left( t \right)+\mu_{j}\left( t \right)+\epsilon^{n} \right)e_{j,2}\left( t \right).$$

$$\frac{{drvv}_{j,1}}{dt}= d_{j-1}\left( t \right)\left( {1-\delta}_{1,j} \right)\left( 1-\theta_{j}^{b}\left( t \right)-\theta_{j}^{d}\left( t \right) \right){rvv}_{j-1,1}\left( t \right)+{\xi^{vv}}_{j}{wvv}_{j,1}\left( t \right)+\lambda_{j}\left( t \right)\left[ k_{1}v_{j,1}\left( t \right)+{\zeta^{vv}}_{j}{wvv}_{j,1}\left( t \right) \right]-\left( d_{j}\left( t \right)+\mu_{j}\left( t \right)+bxz \chi\sigma_{j}+\delta^{vv} \right){rvv}_{j,1}\left( t \right).$$

$$\frac{{drvv}_{j,2}}{dt}= d_{j-1}\left( t \right)\left( {1-\delta}_{1,j} \right)\left[ \left( \theta_{j}^{b}\left( t \right)+\theta_{j}^{d}\left( t \right) \right){rvv}_{j-1,1}\left( t \right)+{rvv}_{j-1,2}\left( t \right) \right]+{\xi^{vv}}_{j}{wvv}_{j,2}\left( t \right)+\lambda_{j}\left( t \right)\left[ k_{2}v_{j,2}\left( t \right)+{\zeta^{vv}}_{j}{wvv}_{j,2}\left( t \right) \right]-\left( d_{j}\left( t \right)+\mu_{j}\left( t \right)+bxz \chi\sigma_{j}+\delta^{vv} \right){rvv}_{j,2}\left( t \right).$$

$$\frac{{dwvv}_{j,1}}{dt}=d_{j-1}\left( t \right)\left( {1-\delta}_{1,j} \right)\left( 1-\theta_{j}^{b}\left( t \right)-\theta_{j}^{d}\left( t \right)-\theta_{j}^{z}\left( t \right) \right){wvv}_{j-1,1}\left( t \right)+\delta^{vv}{rvv}_{j,1}\left( t \right)+\pi_{1}v_{j,1}\left( t \right)-\left( d_{j}\left( t \right)+\mu_{j}\left( t \right)+\chi\sigma_{j}+{\xi^{vv}}_{j}+{\zeta^{vv}}_{j}\lambda_{j}\left( t \right) \right){wvv}_{j,1}\left( t \right).$$

$$\frac{{dwvv}_{j,2}}{dt}=d_{j-1}\left( t \right)\left( {1-\delta}_{1,j} \right)\left( 1-\theta_{j}^{z}\left( t \right) \right){wvv}_{j-1,2}\left( t \right)+d_{j-1}\left( t \right)\left( {1-\delta}_{1,j} \right)\left( \theta_{j}^{b}\left( t \right)+\theta_{j}^{d}\left( t \right) \right){wvv}_{j-1,1}\left( t \right)+\delta^{vv}{rvv}_{j,2}\left( t \right)+\pi_{2}v_{j,2}\left( t \right)-\left( d_{j}\left( t \right)+\mu_{j}\left( t \right)+\chi\sigma_{j}+{\xi^{vv}}_{j}+{\zeta^{vv}}_{j}\lambda_{j}\left( t \right) \right){wvv}_{j,2}\left( t \right).$$

$$\frac{{dwz}_{j}}{dt}=\omega^{z}{vzw}_{j}\left( t \right)+d_{j-1}\left( t \right)\left( {1-\delta}_{1,j} \right)\left[ \left( 1-Tz \right)\theta_{j}^{z}\left( t \right)w_{j-1}\left( t \right)+{wz}_{j-1}\left( t \right) \right]-\left( d_{j}\left( t \right)+\mu_{j}\left( t \right)+\sigma_{j} \right){wz}_{j}\left( t \right).$$

$$\frac{{devb}_{j,1}}{dt}= d_{j-1}\left( t \right)\left( {1-\delta}_{1,j} \right)\left( 1-\theta_{j}^{b}\left( t \right)-\theta_{j}^{d}\left( t \right) \right){evb}_{j-1,1}\left( t \right)+\lambda_{j}\left( t \right){vs}_{j,1}\left( t \right)- \left( d_{j}\left( t \right)+\mu_{j}\left( t \right)+\epsilon^{vb} \right){evb}_{j,1}\left( t \right).$$

$$\frac{{devb}_{j,2}}{dt}= d_{j-1}\left( t \right)\left( {1-\delta}_{1,j} \right){evb}_{j-1,2}\left( t \right)+\lambda_{j}\left( t \right){vs}_{j,2}\left( t \right)+d_{j-1}\left( t \right)\left( {1-\delta}_{1,j} \right)\left( \theta_{j}^{b}\left( t \right)+\theta_{j}^{d}\left( t \right) \right){evb}_{j-1,1}\left( t \right)- \left( d_{j}\left( t \right)+\mu_{j}\left( t \right)+\epsilon^{vb} \right){evb}_{j,2}\left( t \right).$$

$$\frac{{divb}_{j}}{dt}= d_{j-1}\left( t \right)\left( {1-\delta}_{1,j} \right){ivb}_{j-1}\left( t \right)+\epsilon^{vb}\left( {evb}_{j,1}\left( t \right)+{evb}_{j,2}\left( t \right) \right)-\left( d_{j}\left( t \right)+\mu_{j}\left( t \right)+\gamma^{vb}+d_{j}^{vb} \right){ivb}_{j}\left( t \right).$$

$$\frac{{drvb}_{j}}{dt}= d_{j-1}\left( t \right)\left( {1-\delta}_{1,j} \right){rvb}_{j-1}\left( t \right)+\left( {\xi^{vb}}_{j}+{\zeta^{vb}}_{j}\lambda_{j}\left( t \right) \right){wvb}_{j}\left( t \right)+\gamma^{vb}{ivb}_{j}\left( t \right)-\left( d_{j}\left( t \right)+\mu_{j}\left( t \right)+\delta^{vb}+bxz \chi\sigma_{j} \right){rvb}_{j}\left( t \right).$$

$$\frac{{dwvb}_{j}}{dt}= d_{j-1}\left( t \right)\left( {1-\delta}_{1,j} \right)\left( 1-\theta_{j}^{z}\left( t \right) \right){wvb}_{j-1}\left( t \right)+\delta^{vb}{rvb}_{j}\left( t \right)-\left( d_{j}\left( t \right)+\mu_{j}\left( t \right)+\chi\sigma_{j}+{\xi^{vb}}_{j}+{\zeta^{vb}}_{j}\lambda_{j}\left( t \right) \right){wvb}_{j}\left( t \right).$$

$$\frac{{dwvbz}_{j}}{dt}=\omega^{z}{vzb}_{j}\left( t \right)+d_{j-1}\left( t \right)\left( {1-\delta}_{1,j} \right)\left[ \left( 1-Tz \right)\theta_{j}^{z}\left( t \right){wvb}_{j-1}\left( t \right)+{wvbz}_{j-1}\left( t \right) \right]-\left( d_{j}\left( t \right)+\mu_{j}\left( t \right)+\sigma_{j} \right){wvbz}_{j}\left( t \right).$$

$$\frac{{dwvvz}_{j}}{dt}=\omega^{z}{vzv}_{j}\left( t \right)+d_{j-1}\left( t \right)\left( {1-\delta}_{1,j} \right)\left[ \left( 1-Tz \right)\theta_{j}^{z}\left( t \right)\left( {vq}_{j-1,1}\left( t \right)+{vq}_{j-1,2}\left( t \right)+{vs}_{j-1,1}\left( t \right)+{vs}_{j-1,2}\left( t \right)+{wvv}_{j-1,1}\left( t \right)+{wvv}_{j-1,2}\left( t \right) \right)+{wvvz}_{j-1}\left( t \right) \right]-\left( d_{j}\left( t \right)+\mu_{j}\left( t \right)+\chi\sigma_{j} \right){wvvz}_{j}\left( t \right).$$

$$\frac{{dz}_{j}}{dt}= \sigma_{j}\left( bxz r_{j}\left( t \right)+bz {vzw}_{j}\left( t \right)+w_{j}\left( t \right)+{wz}_{j}\left( t \right) \right)+d_{j-1}\left( t \right)\left( {1-\delta}_{1,j} \right)z_{j-1}\left( t \right)-\left( d_{j}\left( t \right)+\mu_{j}\left( t \right)+\eta^{n}+d_{j}^{z} \right)z_{j}\left( t \right).$$

$$\frac{{dzvacc}_{j}}{dt}=d_{j-1}\left( t \right)\left( {1-\delta}_{1,j} \right){zvacc}_{j-1}\left( t \right)+\chi\sigma_{j}\left( bxz {rvv}_{j,1}\left( t \right)+bxz {rvv}_{j,2}\left( t \right)+bz {vzv}_{j}\left( t \right)+{wvv}_{j,1}\left( t \right)+{wvv}_{j,2}\left( t \right)+{wvvz}_{j}\left( t \right) \right)-\left( d_{j}\left( t \right)+\mu_{j}\left( t \right)+\eta^{vv}+d_{j}^{z} \right){zvacc}_{j}\left( t \right).$$

$$\frac{{dzwild}_{j}}{dt}=d_{j-1}\left( t \right)\left( {1-\delta}_{1,j} \right){zwild}_{j-1}\left( t \right)+\chi\sigma_{j}\left( bxz {rvb}_{j}\left( t \right)+bz {vzb}_{j}\left( t \right)+{wvb}_{j}\left( t \right)+{wvbz}_{j}\left( t \right) \right)-\left( d_{j}\left( t \right)+\mu_{j}\left( t \right)+\eta^{vb}+d_{j}^{z} \right){zwild}_{j}\left( t \right).$$

$$\frac{{drz}_{j}}{dt}=d_{j-1}\left( t \right)\left( {1-\delta}_{1,j} \right){rz}_{j-1}\left( t \right)+\eta^{n}z_{j}\left( t \right)+\eta^{vb}{zwild}_{j}\left( t \right)+\eta^{vv}{zvacc}_{j}\left( t \right)-\left( d_{j}\left( t \right)+\mu_{j}\left( t \right) \right){rz}_{j}\left( t \right).$$

$$\frac{{dndv}_{j}}{dt}=d_{j-1}\left( t \right)\left( {1-\delta}_{1,j} \right){ndv}_{j-1}\left( t \right)+d_{j}^{v}i_{j}\left( t \right)+d_{j}^{vb}{ivb}_{j}\left( t \right)-\left( d_{j}\left( t \right)+\mu_{j}\left( t \right) \right){ndv}_{j}\left( t \right).$$

$$\frac{{dndz}_{j}}{dt}=d_{j-1}\left( t \right)\left( {1-\delta}_{1,j} \right){ndz}_{j-1}\left( t \right)+d_{j}^{z}\left( z_{j}\left( t \right)+{zwild}_{j}\left( t \right)+{zvacc}_{j}\left( t \right) \right)-\left( d_{j}\left( t \right)+\mu_{j}\left( t \right) \right){ndz}_{j}\left( t \right).$$

$$\lambda_{j}\left( t \right)=\sum_{a} \beta_{j,a}\left( i_{a}\left( t \right)+\rho^{v}{ivb}^{a}\left( t \right)+\rho^{z}z_{a}\left( t \right)+\rho^{z}{zwild}_{a}\left( t \right)+\rho^{z}{zvacc}_{a}\left( t \right) \right).$$

$$B^{m}\left( t \right)=\Lambda(t)- \frac{\Lambda(t)}{\sum_{j} f_{j}(t)n_{j}}\sum_{j} \left( f_{j}(t)s_{j}\left( t \right)+\sum_{l} f_{j}(t)s_{j,l}\left( t \right) \right).$$

$$B^{s}\left( t \right)= \frac{\Lambda(t)}{\sum_{j} f_{j}(t)n_{j}}\sum_{j} \left( f_{j}(t)s_{j}\left( t \right)+\sum_{l} f_{j}(t)s_{j,l}\left( t \right) \right).$$

$$\Lambda\left( t \right)=U\left( 0,t \right)=\int_{0}^{\infty} f\left( a,t \right)U(a,t)da, t\geq0.$$

$$d_{j}\left( t \right)\equiv\frac{U\left( a_{j},t \right)}{\int_{a_{j-1}}^{a_{j}} U\left( a,t \right)da}$$

$$\begin{matrix} U\left( a,t \right)=\left\{ \begin{matrix} U\left( a-t, 0 \right)\exp\left( -\int_{0}^{t} \varpi(a-t+\tau, \tau) d\tau\right) & t<a \\ \Lambda\left( t-a \right)\left( -\int_{0}^{a} \varpi(\tau,t-a+\tau) d\tau\right) & t\geq a \end{matrix} \right. & \end{matrix}$$

# Calibration Data

## Varicella Seroprevalence

Seroprevalence data from 2017, obtained from [20], were stratified according to age strata given in Table J. For <40-year-olds, seroprevalence mean and standard deviation were estimated by assuming that data were Beta distributed. For ≥40-year-olds, 100% of samples were seropositive.

Table J: Varicella seroprevalence in Belgium

| **Age (years)** | **Seroprevalence** | **Standard Deviation** |
| --- | --- | --- |
| <2 | 36.0% | 0.051 |
| 2-4 | 43.3% | 0.036 |
| 4-6 | 76.8% | 0.032 |
| 6-8 | 86.4% | 0.025 |
| 8-10 | 95.0% | 0.017 |
| 10-12 | 96.1% | 0.014 |
| 12-14 | 93.8% | 0.018 |
| 14-16 | 94.9% | 0.016 |
| 16-18 | 95.0% | 0.016 |
| 18-20 | 95.4% | 0.012 |
| 20-25 | 99.0% | 0.007 |
| 25-30 | 96.3% | 0.014 |
| 30-35 | 99.5% | 0.005 |
| 35-40 | 97.5% | 0.011 |
| 40-50 | 100.0% | N/A |
| 50-60 | 100.0% | N/A |
| ≥60 | 100.0% | N/A |

## Herpes Zoster Incidence

Herpes zoster (HZ) general practice (GP) consultation incidence were used for model calibration under the assumption that 100% of HZ reactivations result in a GP consultation. The number of herpes zoster GP consultations per observed life year were available for 2006-2008 (unstratified by year) [21] and 2012-2018 (stratified by year) [22], see Table K. Mean and standard deviation were computed assuming data are binomially distributed.

Table K: Herpes zoster general practice consultation incidence (2006-2008; 2012-2018)

| **Age (years)** | **Incidence (per 100,000)** | | | | | | | |
| --- | --- | --- | --- | --- | --- | --- | --- | --- |
|  | **2006-2008** | **2012** | **2013** | **2014** | **2015** | **2016** | **2017** | **2018** |
| 0-4 | 140 | 132 | 132 | 160 | 337 | 96 | 244 | 113 |
| 5-9 | 201 | 493 | 476 | 859 | 493 | 417 | 397 | 354 |
| 10-14 | 246 | 776 | 622 | 531 | 627 | 595 | 412 | 411 |
| 15-19 | 243 | 369 | 595 | 563 | 479 | 297 | 253 | 390 |
| 20-24 | 208 | 222 | 409 | 402 | 405 | 386 | 345 | 340 |
| 25-29 | 178 | 200 | 367 | 262 | 484 | 386 | 390 | 312 |
| 30-34 | 169 | 402 | 373 | 357 | 436 | 355 | 224 | 282 |
| 35-39 | 182 | 328 | 513 | 374 | 345 | 248 | 323 | 309 |
| 40-44 | 220 | 385 | 328 | 276 | 429 | 220 | 252 | 184 |
| 45-49 | 285 | 309 | 577 | 652 | 487 | 388 | 565 | 439 |
| 50-54 | 378 | 621 | 551 | 611 | 655 | 521 | 477 | 417 |
| 55-59 | 485 | 798 | 930 | 916 | 825 | 819 | 628 | 521 |
| 60-64 | 583 | 812 | 779 | 789 | 700 | 699 | 922 | 748 |
| 65-69 | 683 | 1106 | 780 | 870 | 1084 | 1021 | 965 | 748 |
| 70-74 | 796 | 1394 | 981 | 1133 | 1461 | 835 | 874 | 781 |
| 75-79 | 952 | 1335 | 1424 | 1237 | 1293 | 1371 | 768 | 892 |
| 80-84 | 1154 | 1213 | 1253 | 1125 | 1226 | 964 | 1317 | 842 |
| ≥85 | 1398 | 1236 | 781 | 964 | 1328 | 1400 | 781 | 975 |

# Economic Model

## Health Utility Outcomes

Healthy quality-adjusted life-year (QALY) values were taken Bouckaert and colleagues [23] (Table L). We assumed QALY loss was 0.004 and 0.005 for natural varicella infections of <15-year-olds and ≥15-year-olds, respectively [24]. We assumed that QALY loss was 0.001 for breakthrough varicella infections for all ages [24].

Table L: Healthy QALY values

| **Age (years)** | **QALY** |
| --- | --- |
| <18 | 1 |
| 18-25 | 0.953 |
| 25-35 | 0.921 |
| 35-45 | 0.920 |
| 45-55 | 0.889 |
| 55-65 | 0.881 |
| 65-75 | 0.848 |
| ≥75 | 0.761 |
| **Source** | **[23]** |

Weights for QALY loss due to herpes zoster were reported by van Hoek and colleagues [25] and were stratified by pain severity: no pain, mild pain, moderate pain, and severe pain. Distribution of HZ cases by pain severity for <70-year-olds and ≥70-year-olds without postherpetic neuralgia (PHN) and for all ages for individuals with PHN. These findings are summarized in Table M. Taking a weighted average yielded QALY weights of 0.9133 and 0.7594 for <70-year-olds and ≥70-year-olds for individuals without PHN and 0.6671 for all ages for individuals with PHN. The proportion of HZ cases that result in PHN were previously estimated by Gauthier and colleagues [26] and were re-aggregated (by averaging values) to conform to the age stratification in this model (Table N).

Table M: QALY weights and distribution of herpes zoster cases by pain severity

| **Pain Severity** | **QALY weights by pain severity** | **Distribution of HZ cases by pain severity** | | |
| --- | --- | --- | --- | --- |
|  |  | **Without PHN** | | **With PHN**  **(All ages)** |
|  |  | **<70 years** | **≥70 years** |  |
| No pain | 1 | 64% | 45% | 0% |
| Mild pain | 0.91 | 24% | 41% | 0% |
| Moderate pain | 0.71 | 4% | 5% | 89% |
| Severe pain | 0.32 | 8% | 9% | 11% |
| **Source** | **[25]** | **[26]** | | |

Table N: Proportion of herpes zoster cases that develop PHN

| **Age (years)** | **Proportion** | **Proportion (aggregated)** |
| --- | --- | --- |
| <50 | 0% | 0% |
| 50-55 | 8% | 9% |
| 55-60 | 10% |  |
| 60-65 | 11% | 12% |
| 65-70 | 13% |  |
| 70-75 | 15% | 17% |
| 75-80 | 18% |  |
| 80-85 | 21% | 20% |
| 85-90 | 19% |  |
| ≥90 |  | 19% |
| **Source** | **[26]** | **Calculated** |

## Vaccination Costs

Costs for pediatric vaccines are recorded in Table O. Pediatric vaccination costs were assumed to comprise the per-dose vaccine costs and a €5 administration fee per administered vaccine. Vaccination with shingles comprised two doses, which cost €170.26 per dose in vaccine costs and €30 per dose in vaccine administration fees. Thus, a full course of shingles vaccine cost a total of (€170.26 + €30) x 2 = €400.52. Indirect vaccination costs (e.g., productivity loss for a caregiver who accompanies a child to a vaccination appointment) were excluded from this analysis.

Table O: Pediatric vaccine costs (2023 Euros)

| **Vaccine** | **Cost per dose (€)** | **Source** |
| --- | --- | --- |
| VARIVAX® (V-MSD) | 52.52 | **[27]** |
| VARILRIX (V-GSK) | 49.44 | **[27]** |
| ProQuad® (MMRV-MSD) | 73.69 | **[27]** |
| PRIORIX-TETRA (MMRV-GSK) | 73.69 | **Assumption (price parity)** |
| M-M-RvaxPro | 25.80 | **[27]** |
| PRIORIX | 22.83 | **[27]** |

## Varicella Costs

### Varicella Direct Costs

Varicella direct costs comprised outpatient visit costs and hospitalization costs.

#### Varicella Outpatient Costs

The fraction of varicella infections that sought GP care was estimated from Riera-Montes and colleagues (Table P). Varicella GP consultations were estimated to cost € 44.25 (2023 Euros, inflated from Bilcke and colleagues [21]).

Table P: Fraction of varicella infections seeking GP care

| **Age (years)** | **Community incidence**  **(per 100,000)** | **GP consultation incidence**  **(per 100,000)** | **Varicella infections**  **seeking GP care** |
| --- | --- | --- | --- |
| <5 | 14,628 | 4,502 | 30.8% |
| 5-10 | 4,126 | 1,006 | 24.4% |
| 10-15 | 284 | 190 | 66.9% |
| 15-20 | 220 | 63 | 28.6% |
| 20-40 | 120 | 49 | 40.8% |
| ≥40 | 38 | 15 | 39.5% |
| **Source** | **[18]** | **[18]** | **Calculated** |

#### Varicella Hospitalization Costs

The fraction of varicella infections that were hospitalized was estimated from Riera-Montes and colleagues (Table Q). Hospitalization costs were estimated to cost € 3,538.29 (2023 Euros, inflated from Blicke and colleagues [21]).

Table Q: Fraction of varicella infections that result in hospitalization

| **Age (years)** | **Community incidence**  **(per 100,000)** | **Hospitalization incidence**  **(per 100,000)** | **Varicella infections**  **Resulting in hospitalization** |
| --- | --- | --- | --- |
| <5 | 14,628 | 79 | 0.54% |
| 5-10 | 4,126 | 3 | 0.07% |
| 10-15 | 284 | 2 | 0.53% |
| 15-20 | 220 | 2 | 0.68% |
| 20-40 | 120 | 2 | 1.67% |
| ≥40 | 38 | 1 | 1.32% |
| **Source** | **[18]** | **[18]** | **Calculated** |

### Indirect Varicella Costs

Varicella indirect costs comprised productivity loss due to varicella infection and varicella hospitalization. The average workdays lost due to varicella infection were previously estimated by Carrico and colleagues to be 0.6 days for <15-year-olds, and 5.7 days for ≥15-year-olds [24, 28, 29]. The average duration of hospitalization was estimated by Bilcke and colleagues to be 5.07 days [30]. Productivity loss for <65-year-olds was assumed to be equal to the loss in wages (we assumed no productivity loss for ≥65-year-olds). The average daily wage was estimated to be € 212.02 (2023 Euros, inflated from [31]).

## Herpes Zoster Costs

### Herpes Zoster Direct Costs

Herpes zoster direct costs comprised GP visit costs and hospitalization costs. The fraction of HZ cases that are hospitalized was estimated from Bilcke and colleagues [21]. We assumed that all HZ cases seek GP care. We further assumed that costs for HZ hospitalizations included any ancillary GP care.

Table R: Herpes zoster outpatient and inpatient resource use

| **Age (years)** | **Fraction of HZ cases  that are hospitalized** | **Fraction of HZ cases that  seek GP and are not hospitalized** |
| --- | --- | --- |
| <5 | 3.40% | 96.60% |
| 5-20 | 0.85% | 99.15% |
| 20-40 | 1.24% | 98.76% |
| 40-50 | 1.73% | 98.27% |
| 50-60 | 2.12% | 97.88% |
| 60-70 | 2.97% | 97.03% |
| 70-80 | 4.36% | 95.64% |
| 80-90 | 7.95% | 92.05% |
| ≥90 | 5.23% | 94.77% |
| **Source** | **[21]** | **Calculated** |

We assumed that whether an individual’s pain level (no pain, mild pain, moderate pain, and severe pain), hospitalized status (hospitalized or not), and PHN status (developed PHN or not) were independent of each other. The joint distribution was then calculated from Table M, Table N, and Table P (Table S). Hospitalization and GP visit costs were then estimated by taking a weighted average of previously reported HZ costs (Table T) [19]. Herpes zoster direct costs are summarized in Table U.

Table S: Joint distribution for herpes zoster hospitalization, PHN status, and pain severity

| **Age (years)** | **With PHN** | | | | **Without PHN** | | | | | | | | **Total** |
| --- | --- | --- | --- | --- | --- | --- | --- | --- | --- | --- | --- | --- | --- |
|  | **Hospitalized** | | **Not hospitalized** | | **Hospitalized** | | | | **Not hospitalized** | | | |  |
|  | **Moderate pain** | **Severe pain** | **Moderate pain** | **Severe pain** | **No pain** | **Mild pain** | **Moderate pain** | **Severe pain** | **No pain** | **Mild pain** | **Moderate pain** | **Severe pain** |  |
| <5 | 0 | 0 | 0 | 0 | 2.19% | 0.81% | 0.13% | 0.27% | 62.17% | 22.96% | 3.83% | 7.65% | 100% |
| 5-20 | 0 | 0 | 0 | 0 | 0.55% | 0.20% | 0.03% | 0.07% | 63.81% | 23.56% | 3.93% | 7.85% | 100% |
| 20-40 | 0 | 0 | 0 | 0 | 0.80% | 0.30% | 0.05% | 0.10% | 63.56% | 23.47% | 3.91% | 7.82% | 100% |
| 40-50 | 0 | 0 | 0 | 0 | 1.11% | 0.41% | 0.07% | 0.14% | 63.24% | 23.35% | 3.89% | 7.78% | 100% |
| 50-60 | 0.17% | 0.02% | 7.84% | 0.97% | 1.24% | 0.46% | 0.08% | 0.15% | 57.32% | 21.16% | 3.53% | 7.05% | 100% |
| 60-70 | 0.32% | 0.04% | 10.36% | 1.28% | 1.68% | 0.62% | 0.10% | 0.21% | 54.95% | 20.29% | 3.38% | 6.76% | 100% |
| 70-80 | 0.64% | 0.08% | 14.04% | 1.74% | 1.64% | 1.49% | 0.18% | 0.33% | 35.94% | 32.74% | 3.99% | 7.19% | 100% |
| 80-90 | 1.42% | 0.17% | 16.38% | 2.03% | 2.86% | 2.61% | 0.32% | 0.57% | 33.14% | 30.19% | 3.68% | 6.63% | 100% |
| ≥90 | 0.88% | 0.11% | 16.03% | 1.98% | 1.91% | 1.74% | 0.21% | 0.38% | 34.54% | 31.47% | 3.84% | 6.91% | 100% |

Table T: Herpes zoster costs by hospitalization status, PHN status, and pain severity (2023 Euros)

| **Age**  **(years)** | **With PHN** | | | | | **Without PHN** | | | | | | | | |
| --- | --- | --- | --- | --- | --- | --- | --- | --- | --- | --- | --- | --- | --- | --- |
|  | **Hospitalized** | | **Not hospitalized** | | **Hospitalized** | | | | | **Not hospitalized** | | | |  |
|  | **Moderate pain** | **Severe pain** | **Moderate pain** | **Severe pain** | **No pain** | | **Mild pain** | **Moderate pain** | **Severe pain** | **No pain** | **Mild pain** | **Moderate pain** | **Severe pain** |  |
| All ages | € 6,638 | € 11,073 | € 487 | € 755 | € 5,699 | | € 5,669 | € 5,914 | € 6,543 | € 30 | € 91 | € 218 | € 221 |  |
| **Source** | **[19]** | | | | | | | | | | | | |  |

Table U: Summary of herpes zoster GP and hospitalization costs by age (2023 Euros)

| **Age (years)** | **GP costs** | **Hospitalization costs** |  |
| --- | --- | --- | --- |
| <5 | € 67.17 | € 5,767.22 |  |
| 5-20 | € 67.17 | € 5,767.22 |  |
| 20-40 | € 67.17 | € 5,767.22 |  |
| 40-50 | € 67.17 | € 5,767.22 |  |
| 50-60 | € 107.57 | € 5,889.52 |  |
| 60-70 | € 121.04 | € 5,930.28 |  |
| 70-80 | € 153.42 | € 5,996.58 |  |
| 80-90 | € 168.62 | € 6,043.93 |  |
| ≥90 | € 164.28 | € 6,030.40 |  |
| **Source** | **Calculated** | | |

### Herpes Zoster Indirect costs

Herpes zoster indirect costs comprised productivity loss due to herpes zoster infection (for those not hospitalized) and herpes zoster hospitalization. Productivity loss for ≥65-year-olds was assumed to be zero. The average number of workdays lost for <65-year-olds with HZ were estimated to be 3.3 days [32] and 7.0 days [21] for non-hospitalized and hospitalized HZ cases, respectively. The cost of productivity loss was equal to the workdays lost multiplied by the average daily wage, which was previously estimated to be € 212.02 (2023 Euros, inflated from [31]).

# Vaccine Parameterization

## Varicella Vaccine

Varicella vaccination followed the parameterization of Sharomi and colleagues (see Supplementary Materials Section S4 of [8]), which was based on the study by Pillsbury and colleagues [33].

## Herpes Zoster Vaccine

We assumed that herpes zoster vaccination confers perfect temporary immunity that waned at rate $\omega z$. We simulated a herpes zoster vaccine trial among ≥50-year-olds (age strata $j\geq45$) using our model. Initial conditions and parameter values are taken from the calibrated model.

**No-vaccine equations:**

$$r_{j}^{'}\left( t \right)=d_{j-1}\times r_{j-1}\left( t \right)\times\mathbb{I}_{\left\{ j>45 \right\}}+\zeta^{n}\times\lambda_{j}\left( 2022 \right)\times w_{j}\left( t \right)-\left( \delta^{n}+d_{j}+\mu_{j} \right)\times r_{j}\left( t \right)$$

$$w_{j}^{'}\left( t \right)=d_{j-1}\times w_{j-1}\left( t \right)\times\mathbb{I}_{\left\{ j>45 \right\}}+\delta^{n}\times r_{j}\left( t \right)-\left( \zeta^{n}\times\lambda_{j}\left( 2022 \right)+\sigma_{j}+d_{j}+\mu_{j} \right)\times w_{j}\left( t \right)$$

$$z_{j}^{'}\left( t \right)=\sigma_{j}\times w_{j}\left( t \right)$$

**Herpes zoster vaccine equations:**

$$vzw_{j}^{'}\left( t \right)=d_{j-1}\times vzw_{j-1}\left( t \right)\times\mathbb{I}_{\left\{ j>45 \right\}}-\left( \omega z+d_{j}+\mu_{j} \right)\times vzw_{j}\left( t \right)$$

$$wz_{j}^{'}\left( t \right)=d_{j-1}\times wz_{j-1}\left( t \right)\times\mathbb{I}_{\left\{ j>45 \right\}}+\omega z\times vzw_{j}\left( t \right)-\left( \sigma_{j}+d_{j}+\mu_{j} \right)\times wz_{j}\left( t \right)$$

$$z_{j}^{'}\left( t \right)=\sigma_{j}\times wz_{j}\left( t \right)$$

The parameter $\omega z$ was estimated by calibrating the model to data from Boutry and colleagues [34] (see Table V) using the objective function

$$\sum\left( 1-\frac{HZ cases \left( vaccine \right)}{HZ cases \left( no vaccine \right)}-\left( observed vaccine efficacy \right) \right)^{2} .$$

Table V: Herpes zoster vaccine efficacy

| **Year since vaccination** | **Vaccine efficacy (95% confidence interval)** |
| --- | --- |
| 1 | 0.977 (0.931, 0.995) |
| 2 | 0.927 (0.862, 0.966) |
| 3 | 0.924 (0.850, 0.966) |
| 4 | 0.898 (0.803, 0.952) |
| 6 | 0.849 (0.704, 0.931) |
| 7 | 0.853 (0.713, 0.933) |
| 8 | 0.841 (0.644, 0.940) |
| **Source** | **[34]** |

The point estimate of $\omega z$ was 0.009675 per year, equivalent to an average duration of protection of 103.4 years. The posterior distribution of $\omega z$ was estimated by using a bootstrapping procedure. Specifically, we fitted the model to 1,000 realizations of synthetic data that were generated by assuming data (Table V) were beta distributed (beta parameters were chosen to preserve the mean and 95% confidence interval). Estimates of $\omega z$ are shown in Fig D. The posterior distribution was approximately gamma distributed with parameters $\alpha=33.4279$ and $\beta=149.768$. Thus, the 95% confidence region for $\omega z$ was (0.006677, 0.013219). Equivalently, the 95% confidence region for the duration of protection is (75.65, 147.77).


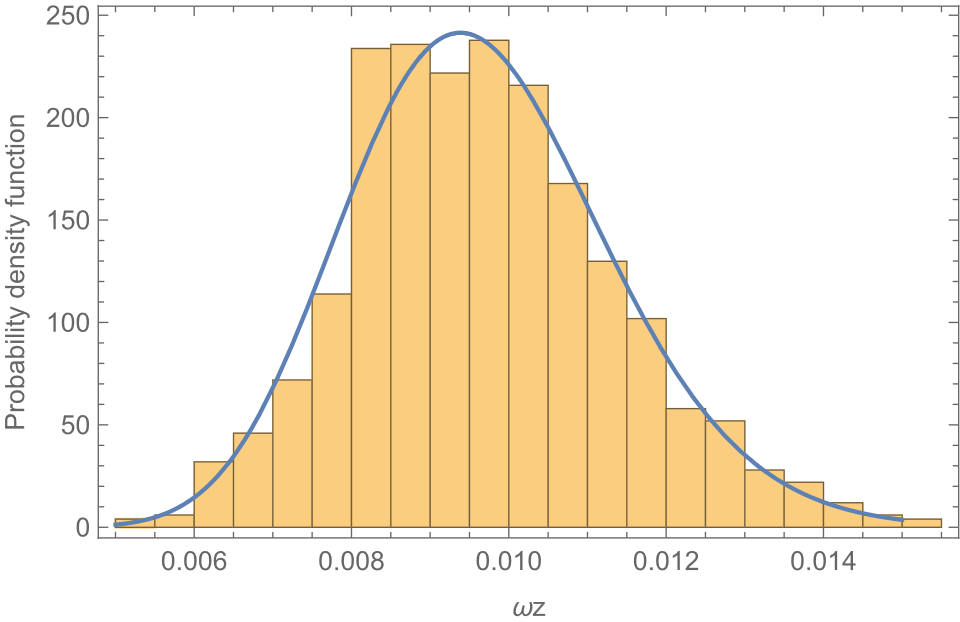


Fig D: Bootstrap estimates of herpes zoster vaccine waning parameter ($\omega z$)

**(Yellow bars) Histogram. (Blue line) Gamma distribution (**$\boldsymbol{\alpha=33.4279}$**,** $\boldsymbol{\beta=149.768}$**)**

The calibrated herpes zoster vaccination model output is plotted against the vaccine efficacy data in Fig E.


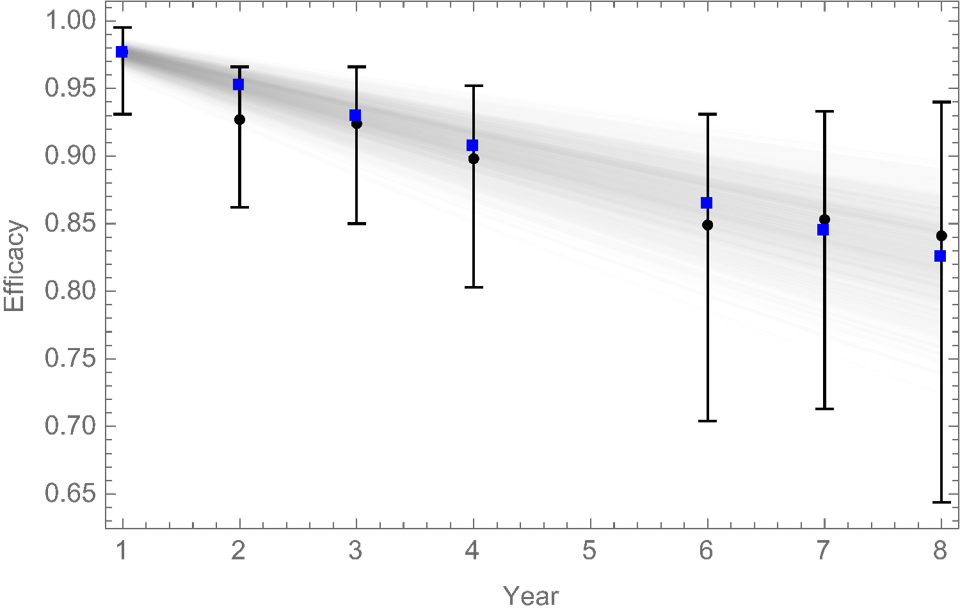


Fig E: Herpes zoster vaccine efficacy

**(Black dots) Efficacy data. (Black bars) 95% confidence interval. (Blue square) Model efficacy estimates for point estimate** $\boldsymbol{\omega z=0.009675}$ **per year. (Grey) Model efficacy estimates for bootstrapped values of** $\boldsymbol{\omega z}$**.**

# Vaccination Strategies

In addition to the HZ-only reference strategy, we considered 13 additional strategies, which are summarized in Table W. Varicella vaccines in Strategy 6 were MMRV-GSK, whereas varicella vaccines in all other strategies were either V-MSD or MMRV-MSD. All varicella vaccinations that occurred at 1 and 8 years of age were assumed to be quadrivalent formulations (i.e., MMRV-MSD or MMRV-GSK), whereas varicella vaccinations at other ages are assumed to be monovalent formulations (i.e., V-MSD). For both varicella and shingles vaccines, catchup programs in all strategies had a duration of 1 year. Vaccine Strategies 1-12 are defined with respect to Strategy 0:

- Strategy 0: Base case vaccination strategy
  - 2-dose routine varicella vaccination with MMRV-MSD at ages 1 (vaccine coverage rage [VCR]: 95%) and 8 (VCR: 90%)
  - 1-dose catchup varicella vaccination program at age 8 with MMRV-MSD for 1 year (VCR: 70%)
  - Routine shingles vaccination at age 60 (VCR: 50%)
- Strategy 1: Lower first- and second-dose routine varicella vaccine coverage
  - Routine first-dose VCR: 90%
  - Routine second-dose VCR: 85%
- Strategy 2: Earlier first dose varicella catchup
  - First dose catchup varicella with V-MSD at age 5 (VCR: 70%)
- Strategy 3: Shingles catchup vaccination program
  - Shingles catchup vaccination for 60-85-year-olds (VCR: 50%)
- Strategy 4: No varicella catchup program
- Strategy 5: 2-dose varicella catchup program
  - Second dose catchup with V-MSD at 12 years (VCR: 65%)
- Strategy 6: Vaccination with MMRV-GSK
- Strategy 7: Later first dose for routine varicella vaccination
  - First dose routine varicella vaccination with V-MSD at age 2 (VCR: 95%)
- Strategy 8: Later first dose for routine varicella vaccination with lower routine coverage
  - First dose routine varicella vaccination with V-MSD at age 2 (VCR: 90%)
  - Routine second-dose VCR: 85%
- Strategy 9: Earlier second dose for routine varicella vaccination
  - Second dose routine varicella vaccination with V-MSD at age 5 (VCR: 90%)
- Strategy 10: Later first and earlier second dose for routine varicella vaccination
  - First dose routine varicella vaccination with V-MSD at age 2 (VCR: 95%)
  - Second dose routine varicella vaccination with V-MSD at age 5 (VCR: 90%)
- Strategy 11: Later first dose varicella catchup
  - First dose catchup varicella with V-MSD at age 12 (VCR: 70%)

In contrast, Strategy 12 consisted of routine two-dose shingles vaccination at 60 years (VCR = 50%) and catch-up two-dose shingles vaccination for 60-85-year-olds (VCR = 50%, 1-year program duration).

Table W: Vaccination strategies

| **Strategy** | **Vaccination Age (years)** | | | | | | **Vaccine coverage** | | | | | |
| --- | --- | --- | --- | --- | --- | --- | --- | --- | --- | --- | --- | --- |
|  | **Varicella** | | | | **Shingles** | | **Varicella** | | | | **Shingles** | |
|  | **Primary** | | **Booster** | | **Routine** | **Catchup** | **Primary** | | **Booster** | | **Routine** | **Catchup** |
|  | **Routine** | **Catchup** | **Routine** | **Catchup** |  |  | **Routine** | **Catchup** | **Routine** | **Catchup** |  |  |
| Reference | N/A | N/A | N/A | N/A | 60 | N/A | N/A | N/A | N/A | N/A | 50% | N/A |
| 0 | 1 | 8 | 8 | N/A | 60 | N/A | 95% | 70% | 90% | N/A | 50% | N/A |
| 1 | 1 | 8 | 8 | N/A | 60 | N/A | 90% | 70% | 85% | N/A | 50% | N/A |
| 2 | 1 | 5 | 8 | N/A | 60 | N/A | 95% | 70% | 90% | N/A | 50% | N/A |
| 3 | 1 | 8 | 8 | N/A | 60 | 60-85 | 95% | 70% | 90% | N/A | 50% | 50% |
| 4 | 1 | N/A | 8 | N/A | 60 | N/A | 95% | N/A | 90% | N/A | 50% | N/A |
| 5 | 1 | 8 | 8 | 12 | 60 | N/A | 95% | 70% | 90% | 65% | 50% | N/A |
| 6* | 1 | 8 | 8 | N/A | 60 | N/A | 95% | 70% | 90% | N/A | 50% | N/A |
| 7 | 2 | 8 | 8 | N/A | 60 | N/A | 95% | 70% | 90% | N/A | 50% | N/A |
| 8 | 2 | 8 | 8 | N/A | 60 | N/A | 90% | 70% | 85% | N/A | 50% | N/A |
| 9 | 1 | 8 | 5 | N/A | 60 | N/A | 95% | 70% | 90% | N/A | 50% | N/A |
| 10 | 2 | 8 | 5 | N/A | 60 | N/A | 95% | 70% | 90% | N/A | 50% | N/A |
| 11 | 1 | 12 | 8 | N/A | 60 | N/A | 95% | 70% | 90% | N/A | 50% | N/A |
| 12 | N/A | N/A | N/A | N/A | 60 | 60-85 | N/A | N/A | N/A | N/A | 50% | 50% |
| Unless otherwise stated varicella vaccination is comprised of V-MSD or MMRV-MSD vaccines | | | | | | | | | | | | |
| * Varicella vaccination is comprised of MMRV-GSK vaccines | | | | | | | | | | | | |

# Sensitivity Analyses

Distributions for vaccine parameters (e.g., take rate, waning rate, etc.) were as described by Lang and colleagues (see Sections S1.6.4-S1.6.9, reproduced below) [35]. Vaccine coverage rates were assumed to be beta distributed with mean equal to the base case value (see Table W) and with standard deviation equal to 5% of the mean. Remaining parameters included in the deterministic and probabilistic sensitivity analyses are summarized in Table X.

Table X: Parameter distributions for sensitivity analyses

| **Symbol** | **Description** | **Mean** | **Standard deviation** | **Distribution** |
| --- | --- | --- | --- | --- |
| **Natural Varicella** | | | | |
| $\omega m$ | Maternal immunity waning rate (per year) | 2 | 10% | Lognormal |
| $\zeta n$ | Fraction of contacts that lead to exogenous boosting | 0.3345 | 20% | Beta |
| $\delta n$ | Waning rate for high HZ immunity state (following natural VZV infection) (per year) | 0.01230 | 20% | Lognormal |
| $\eta n$ | Recovery rate for HZ (per year) | 13.04 | 20% | Lognormal |
| **Breakthrough Varicella** | | | | |
| $\delta vb$ | Waning rate for high HZ immunity state (following breakthrough VZV infection) (per year) | 0.01230 | 20% | Lognormal |
| $\zeta vb$ | Fraction of contacts that lead to exogenous boosting | 0.3345 | 20% | Beta |
| $\eta vb$ | Recovery rate for HZ (per year) | 13.04 | 20% | Lognormal |
| **Varicella Vaccination** | | | | |
| $\delta vv$ | Waning rate for high HZ immunity state (following VZV vaccination) (per year) | 0.01230 | 20% | Lognormal |
| $\zeta vv$ | Fraction of contacts that lead to exogenous boosting | 0.3345 | 20% | Beta |
| $\eta vv$ | Recovery rate for HZ (per year) | 13.04 | 20% | Lognormal |
| $\rho$ | Relative infectiousness of vaccinated individuals | 0.5 | 20% | Beta |
| $\chi$ | Relative reactivation rate for HZ (in VZV or HZ vaccinated individuals) | 0.1667 | 20% | Beta |
| **Herpes Zoster** | | | | |
| $\rho z$ | Relative infectiousness of HZ | 0.07 | 20% | Beta |
| $dphn$ | Duration of postherpetic neuralgia (years) | 0.75 | 20% | Lognormal |
| **Herpes Zoster Vaccination** | | | | |
| $\omega z$ | Waning rate for HZ vaccine protection | See Section 5.2 | | Gamma |
| **Scaling Factors** | | | | |
| $ScaleDoseCostV$ | Scaling factor for direct VZV vaccination costs | 1 | 10% | Lognormal |
| $ScaleCareCostVDirect$ | Scaling factor for direct VZV treatment costs | 1 | 10% | Lognormal |
| $ScaleCareCostVIndirect$ | Scaling factor for indirect VZV treatment costs | 1 | 10% | Lognormal |
| $ScaleDoseCostHZ$ | Scaling factor for direct HZ vaccination costs | 1 | 10% | Lognormal |
| $ScaleCareCostHZDirect$ | Scaling factor for direct HZ treatment costs | 1 | 10% | Lognormal |
| $ScaleCareCostHZIndirect$ | Scaling factor for indirect HZ treatment costs | 1 | 10% | Lognormal |
| $ScaleNVQALYs$ | Scaling factor for natural varicella QALYs | 1 | 5% | Scaled beta |
| $ScaleBVQALYs$ | Scaling factor for breakthrough varicella QALYs | 1 | 5% | Scaled beta |
| $ScaleHZQALYs$ | Scaling factor for HZ QALYs (without PHN) | 1 | 5% | Scaled beta |
| $ScalePHNQALYs$ | Scaling factor for HZ QALYs (with PHN) | 1 | 5% | Scaled beta |

**Sections S1.6.4-S1.6.9 of [35]:**

**S1.6.4, Vaccine Parameters:** Vaccine parameters included in the sensitivity analysis comprise the vaccine failure rate ($P$), vaccine take rates ($T1$ – first dose; $T2$ – second dose), waning of temporary immunity ($\sigma^{v}$), waning of durable immunity ($\Pi=\pi1=\pi2$), and relative infectiousness of breakthrough varicella infections ($\rho^{v}$).

**S1.6.5, Vaccine Failure Rate:** We assume that the successful vaccination rate (P=1-vaccine failure rate) is Beta distributed with mean equal to the base case parameter value and with standard deviation equal to 5% of the base case parameter value.

**S1.6.6, Vaccine take-rates:** We introduce the notation $X2$ to represent the total take rate following both vaccine doses. We assume that the variables $T1$ (dose-1 take rate) and $X2-T1$ (difference between total take rate and dose-1 take rate) are Dirichlet distributed with parameters $a\times b$, $a\times(c-b)$, and $a\times(1-c)$. Here, $b$ is the base case value for $T1$, $c$ is the base case value for $X2$, and a is determined through optimization. Given the above assumptions, we observe that $T1$ is distributed as a Beta distribution with parameters $a\times b$ and $a\times(1-b)$ and $X2$ is distributed as a Beta distribution with parameters $a\times c$ and $a\times(1-c)$. Thus, we choose $a$ to minimize the objective function

$$\left( \sqrt{Variance\left[ Beta\left[ a\times b,a\times\left( 1-b \right) \right] \right]}-\frac{diff^{95}(1)}{2\times1.96} \right)^{2}+\left( \sqrt{Variance[Beta[a\times c,a\times(1-c)]]}-\frac{diff^{95}\left( 2 \right)}{2\times1.96} \right)^{2} ,$$

where $diff^{95}(1)$ is the difference between upper and lower bounds for the 95% confidence interval of $T1$ and $diff^{95}(2)$ is the difference between the upper and lower bounds for the 95% confidence interval of $X2$. We use estimates of $T1$ and $X2$ (MSD: 0.903 [0.878,0.929] and 0.970 [0.952,0.988]; GSK: 0.617 [0.582,0.653] and 0.938 [0.922,0.954], respectively) from Pillsbury, et al. [33]. Estimates of the mean of $T1$ and $X2$ are for MSD products were 0.903 and 0.067, respectively, and estimates of the standard deviation were 0.014 and 0.012. Estimates of the mean of $T1$ and $X2$ are for GSK products were 0.617 and 0.321, respectively, and estimates of the standard deviation were 0.018 and 0.017.

To draw a parameter realization for T1 and T2 we generate the random variables

$$\left( T1,X2-T1 \right)\sim Dirichlet\left[ a\times b,a\times\left( c-b \right),a\times\left( 1-c \right) \right]$$

and then compute

$$\left( T1,T2 \right)=\left( T1, \frac{X2-T1}{1-X1} \right) .$$

**S1.6.7, Waning of temporary immunity:** The 95% CI estimates of the waning rate for temporary immunity ($\sigma v$) for MSD and GSK products are roughly symmetrical. For example, for MSD products $\sigma v$ is estimated to be 0.826 with 95% CI (0.351,1.299), i.e, $\sigma v\approx0.826\pm0.474$; analogously for GSK products, $\sigma v\approx1.136\pm0.308$ [33]. Thus, we model $\sigma v$ as a truncated Normal random variable with support $(0,\infty)$.

**S1.6.8, Waning of durable immunity:** We assume the same value for the waning of durable immunity for MSD and GSK products following either the first or second dose, i.e., $\Pi=\pi1=\pi2$. We further assume that $\pi$ is lognormally distributed with mean equal to the base case value (0.012, i.e., the duration was assumed to be 81.3 years, see [8, 14]) and with standard deviation equal to 20% of the base case value.

**S1.6.9, Relative infectiousness of breakthrough varicella:** We assume that the relative infectiousness of varicella ($\rho^{v}$) is the same for both MSD and GSK products. We further assume that this parameter is distributed as a Beta random variable with mean given by the base case parameter value and with standard deviation equal to 20% of the base case parameter value.

## Scaling Factors

To reduce the dimensionality of the sensitivity analyses, the effect of multiple parameters were aggregated together through the use of a scaling factor.

### Costs

Scaling factors for cost parameters were assumed to be lognormally distributed with mean 1 and standard deviation 0.1. We summarize multipliers for cost parameters below.

**ScaleDoseCostV:** Per-dose varicella vaccine acquisition and administration direct costs were multiplied by the scaling factor *ScaleDoseCostV*.

**ScaleCareCostVDirect:** Per-infection varicella direct treatment costs were multiplied by the scaling factor *ScaleCareCostVDirect*.

**ScaleCareCostVIndirect:** Per-infection varicella indirect treatment costs were multiplied by the scaling factor *ScaleCareCostVDirect*.

**ScaleDoseCostHZ:** Per-dose herpes zoster vaccine acquisition and administration direct costs were multiplied by the scaling factor *ScaleDoseCostHZ***.**

**ScaleCareCostHZDirect:** Per-infection herpes zoster direct treatment costs were multiplied by the scaling factor *ScaleCareCostHZDirect*.

**ScaleCareCostHZIndirect:** Per-infection herpes zoster indirect treatment costs were multiplied by the scaling factor *ScaleCareCostHZIndirect*.

### Quality-adjusted life-years (QALY)

We summarize multipliers for QALY parameters below.

**ScaleNVQALYs:** QALYs for natural varicella infected individuals were multiplied by the scaling factor *ScaleNVQALYs*. *ScaleNVQALYs* was assumed to have the distribution *X*/0.7914, where *X* was beta distributed with mean 0.7914 and standard deviation 0.7914*5%.

**ScaleBVQALYs:** QALYs for breakthrough varicella infected individuals were multiplied by the scaling factor *ScaleBVQALYs*. *ScaleBVQALYs* was assumed to have the distribution *X*/0.9189, where *X* was beta distributed with mean 0.9189 and standard deviation 0.9189*5%.

**ScaleHZQALYs:** QALYs for individuals with herpes zoster (without PHN) were multiplied by the scaling factor *ScaleHZQALYs*. *ScaleHZQALYs* was assumed to have the distribution *X*/0.9133, where *X* was beta distributed with mean 0.9133 and standard deviation 0.9133*5%.

**ScalePHNQALYs:** QALYs for individuals with herpes zoster (with PHN) were multiplied by the scaling factor *ScalePHNQALYs*. *ScalePHNQALYs* was assumed to have the distribution *X*/0.6671, where *X* was beta distributed with mean 0.6671 and standard deviation 0.6671*5%.

# Additional Results

## Model Calibration

### Demographic Model


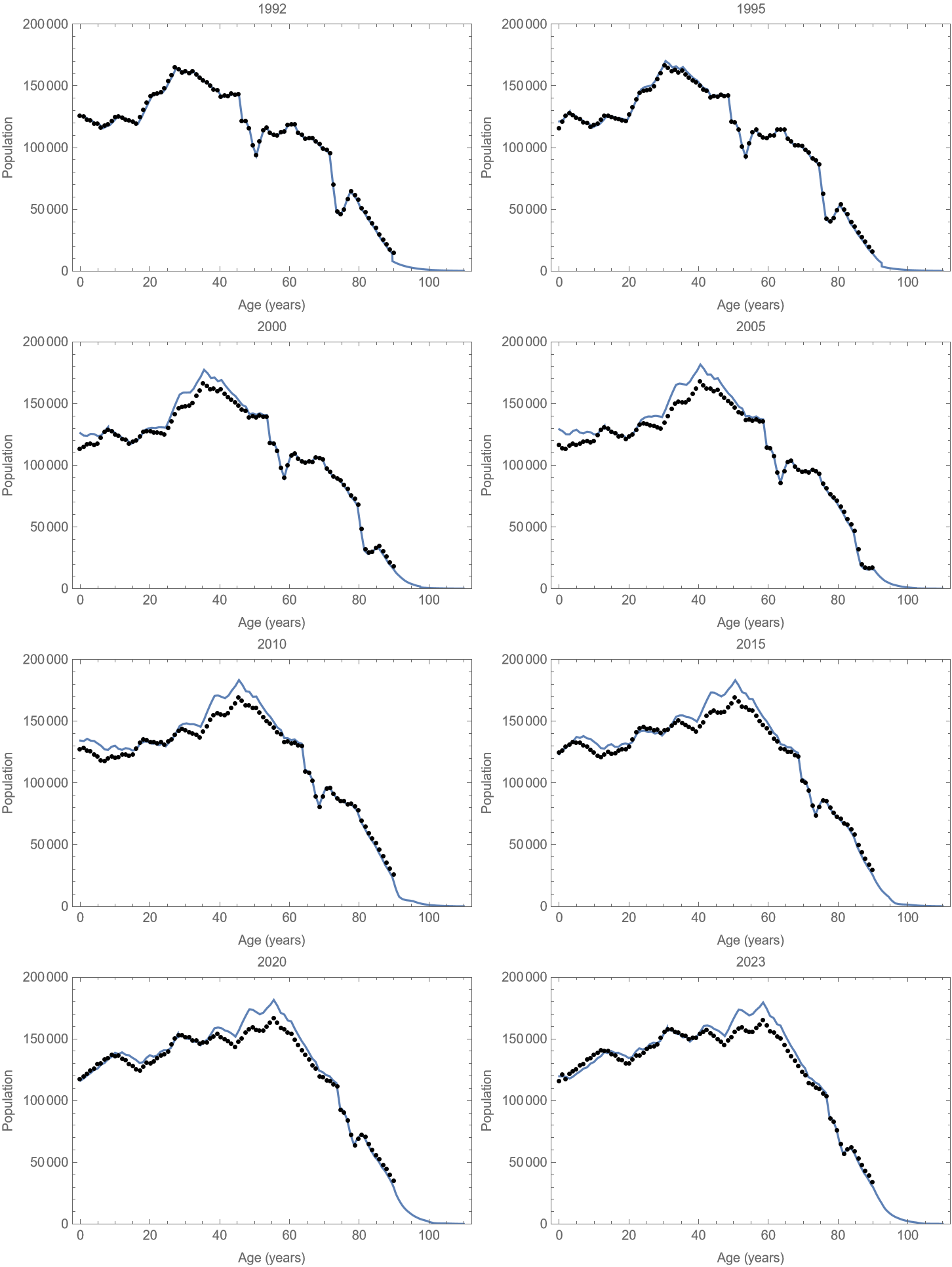


Fig F: Demographic structure (model output versus calibration data, 1992-2023)


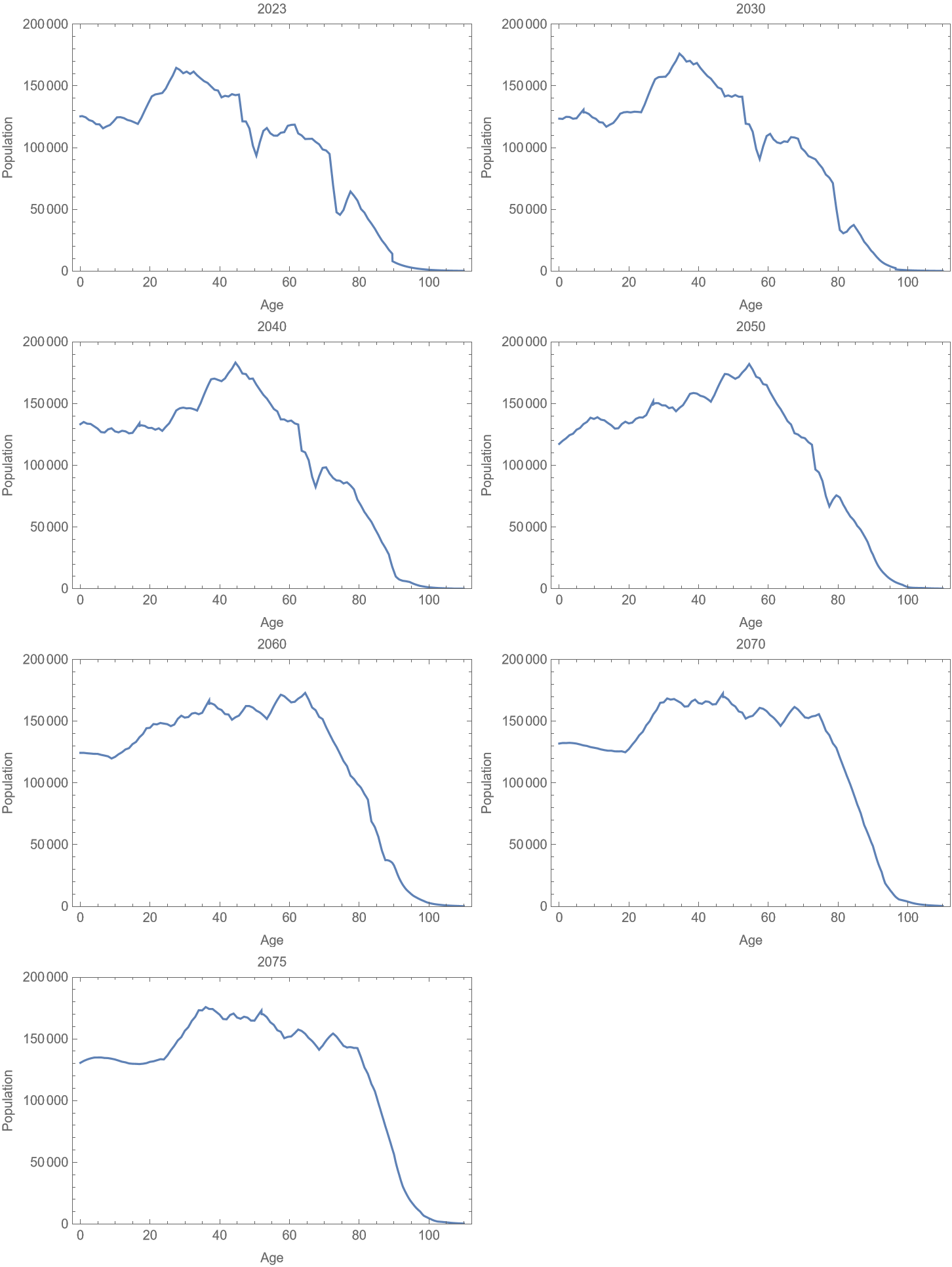


Fig G: Demographic structure (model projection, 2023-2075)

### Epidemiological Model


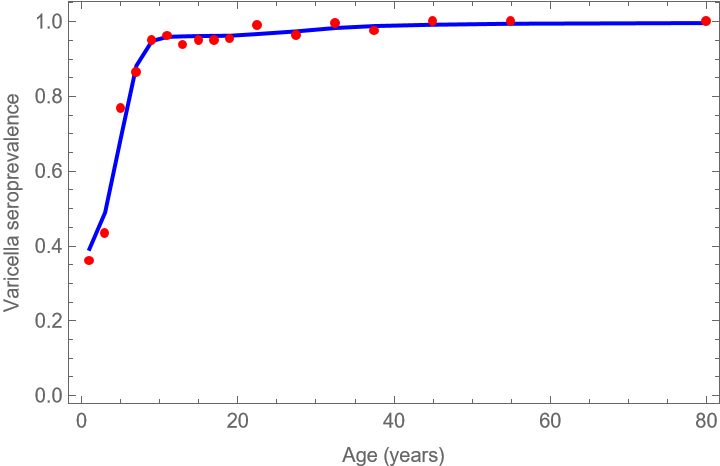


Fig H: Pre-UVV varicella seroprevalence (2017)

**(Dots) Calibration data. (Line) Model output.**

**
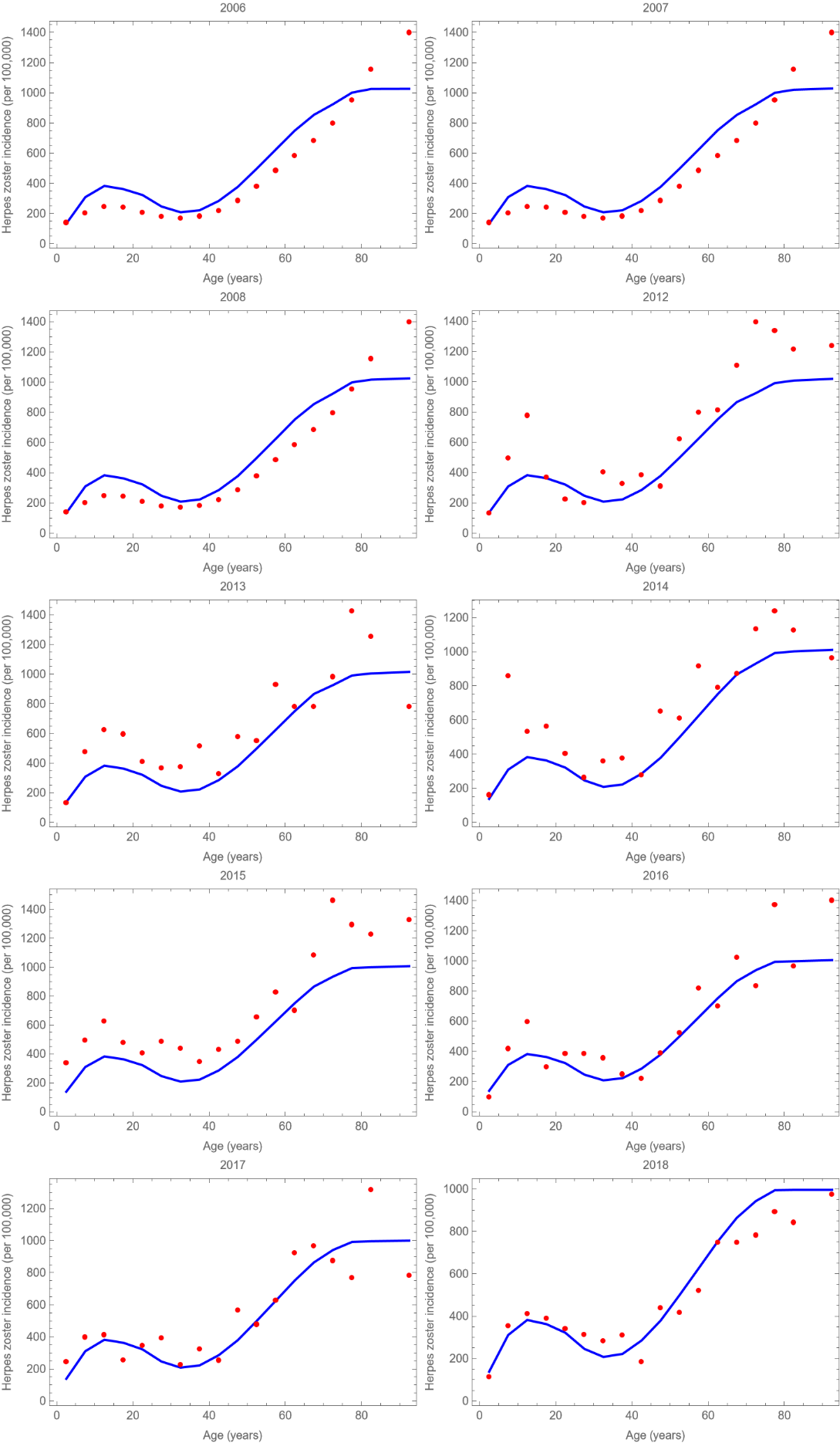
**

Fig I: Herpes zoster incidence (2006-2008 and 2012-2018)

**(Dots) Calibration data. (Line) Model output.**

## Clinical and Economic Outcomes

Table Y: Cumulative clinical outcomes (2023-2073)

| **Strategy** | **Varicella cases** | **Varicella outpatient cases** | **Varicella inpatient cases** | **Varicella deaths** | **Herpes zoster cases** | **Herpes zoster deaths** |
| --- | --- | --- | --- | --- | --- | --- |
| Reference Strategy | 6,525,904 | 1,905,853 | 27,924 | 61 | 3,044,798 | 467 |
| 0 | 585,523 | 181,694 | 3,094 | 24 | 2,944,787 | 483 |
| 1 | 796,077 | 245,258 | 4,098 | 30 | 2,937,853 | 482 |
| 2 | 559,253 | 174,853 | 3,038 | 24 | 2,941,367 | 483 |
| 3 | 583,534 | 181,094 | 3,084 | 24 | 2,805,812 | 442 |
| 4 | 592,537 | 183,931 | 3,141 | 24 | 2,945,025 | 483 |
| 5 | 585,457 | 181,668 | 3,094 | 24 | 2,944,794 | 483 |
| 6 | 1,070,662 | 317,132 | 3,631 | 25 | 2,912,880 | 482 |
| 7 | 709,489 | 221,443 | 3,865 | 25 | 2,934,020 | 482 |
| 8 | 1,017,029 | 313,993 | 5,323 | 31 | 2,930,619 | 481 |
| 9 | 524,593 | 166,094 | 3,047 | 24 | 2,947,369 | 483 |
| 10 | 616,152 | 196,463 | 3,695 | 24 | 2,935,913 | 482 |
| 11 | 590,135 | 183,004 | 3,109 | 24 | 2,945,281 | 483 |
| 12 | 6,525,720 | 1,905,763 | 27,921 | 61 | 2,913,225 | 429 |

Table Z: Marginal cost and QALY outcomes (2023-2073)

| **Strategy** | **Marginal QALYs** | **Marginal costs** | | **ICER** | |
| --- | --- | --- | --- | --- | --- |
|  |  | **Payer perspective** | **Societal perspective** | **Payer perspective** | **Societal perspective** |
| Reference Strategy | 0 | € 0 | € 0 |  |  |
| 0 | 15,035 | € 169,298,010 | -€ 17,524,487 | € 11,260 | -€ 1,166 |
| 1 | 14,602 | € 156,549,926 | -€ 21,219,153 | € 10,721 | -€ 1,453 |
| 2 | 15,154 | € 170,145,220 | -€ 18,434,427 | € 11,227 | -€ 1,216 |
| 3 | 20,508 | € 500,889,315 | € 305,204,726 | € 24,424 | € 14,882 |
| 4 | 15,007 | € 168,948,517 | -€ 17,155,884 | € 11,258 | -€ 1,143 |
| 5 | 15,035 | € 169,383,605 | -€ 17,447,075 | € 11,266 | -€ 1,160 |
| 6 | 14,708 | € 179,825,705 | -€ 5,063,962 | € 12,226 | -€ 344 |
| 7 | 14,838 | € 194,590,408 | € 7,582,875 | € 13,115 | € 511 |
| 8 | 14,173 | € 179,408,518 | € 5,273,599 | € 12,659 | € 372 |
| 9 | 15,118 | € 208,898,489 | € 21,685,060 | € 13,818 | € 1,434 |
| 10 | 14,988 | € 234,701,607 | € 46,172,535 | € 15,659 | € 3,081 |
| 11 | 15,014 | € 169,220,787 | -€ 17,285,843 | € 11,271 | -€ 1,151 |
| 12 | 5,202 | € 333,723,967 | € 325,241,683 | € 64,156 | € 62,525 |

## Sensitivity analyses


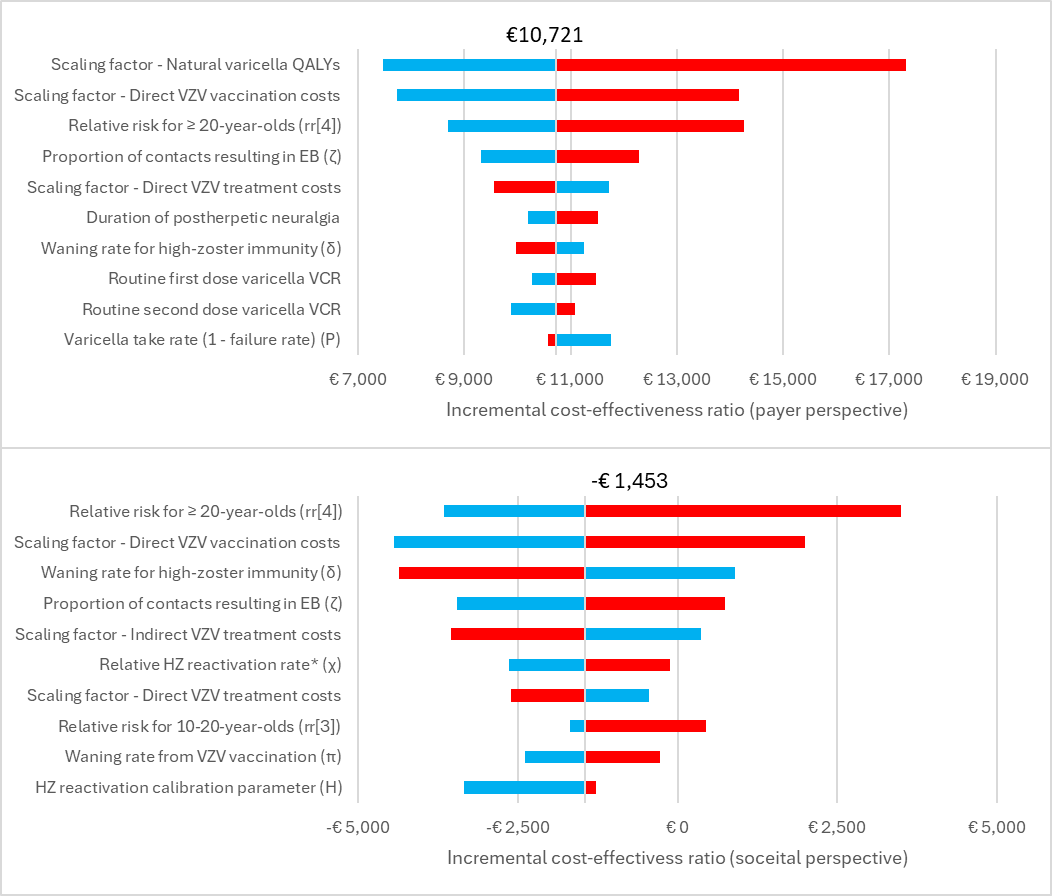


Fig J: Tornado diagram for Strategy 1 incremental cost-effectiveness deterministic sensitivity analysis (DSA) results

**(Blue bars) Parameter lower bound. (Red bars) Parameter upper bound. (Top) Payer perspective. (Bottom) Societal perspective. EB: exogenous boosting; HZ: herpes zoster; QALY: quality-adjusted life-year; VCR: vaccine coverage rate; VZV: varicella zoster virus. * Relative reactivation rate for VZV and HZ vaccinated individuals with respect to reactivation rate of individuals who were infected with natural varicella.**


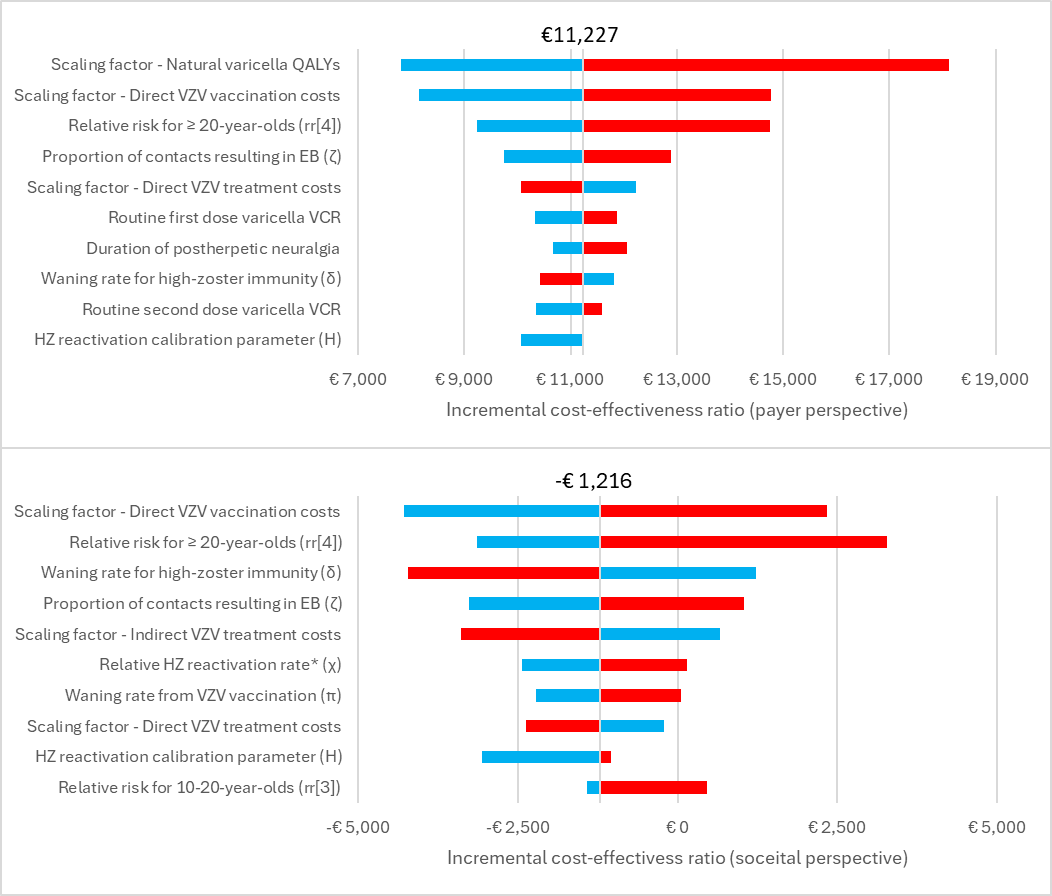


Fig K: Tornado diagram for Strategy 2 incremental cost-effectiveness deterministic sensitivity analysis (DSA) results

**(Blue bars) Parameter lower bound. (Red bars) Parameter upper bound. (Top) Payer perspective. (Bottom) Societal perspective. EB: exogenous boosting; HZ: herpes zoster; QALY: quality-adjusted life-year; VCR: vaccine coverage rate; VZV: varicella zoster virus. * Relative reactivation rate for VZV and HZ vaccinated individuals with respect to reactivation rate of individuals who were infected with natural varicella.**


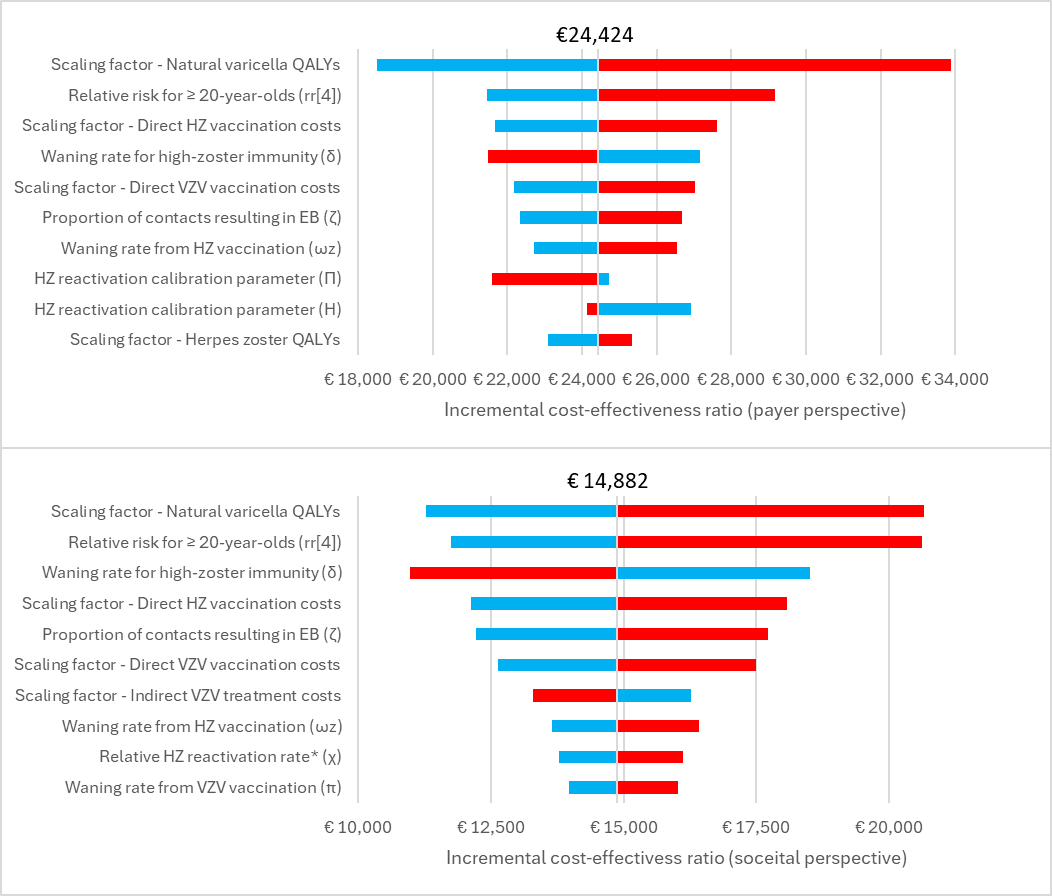


Fig L: Tornado diagram for Strategy 3 incremental cost-effectiveness deterministic sensitivity (DSA) results

**(Blue bars) Parameter lower bound. (Red bars) Parameter upper bound. (Top) Payer perspective. (Bottom) Societal perspective. EB: exogenous boosting; HZ: herpes zoster; QALY: quality-adjusted life-year; VCR: vaccine coverage rate; VZV: varicella zoster virus. * Relative reactivation rate for VZV and HZ vaccinated individuals with respect to reactivation rate of individuals who were infected with natural varicella.**


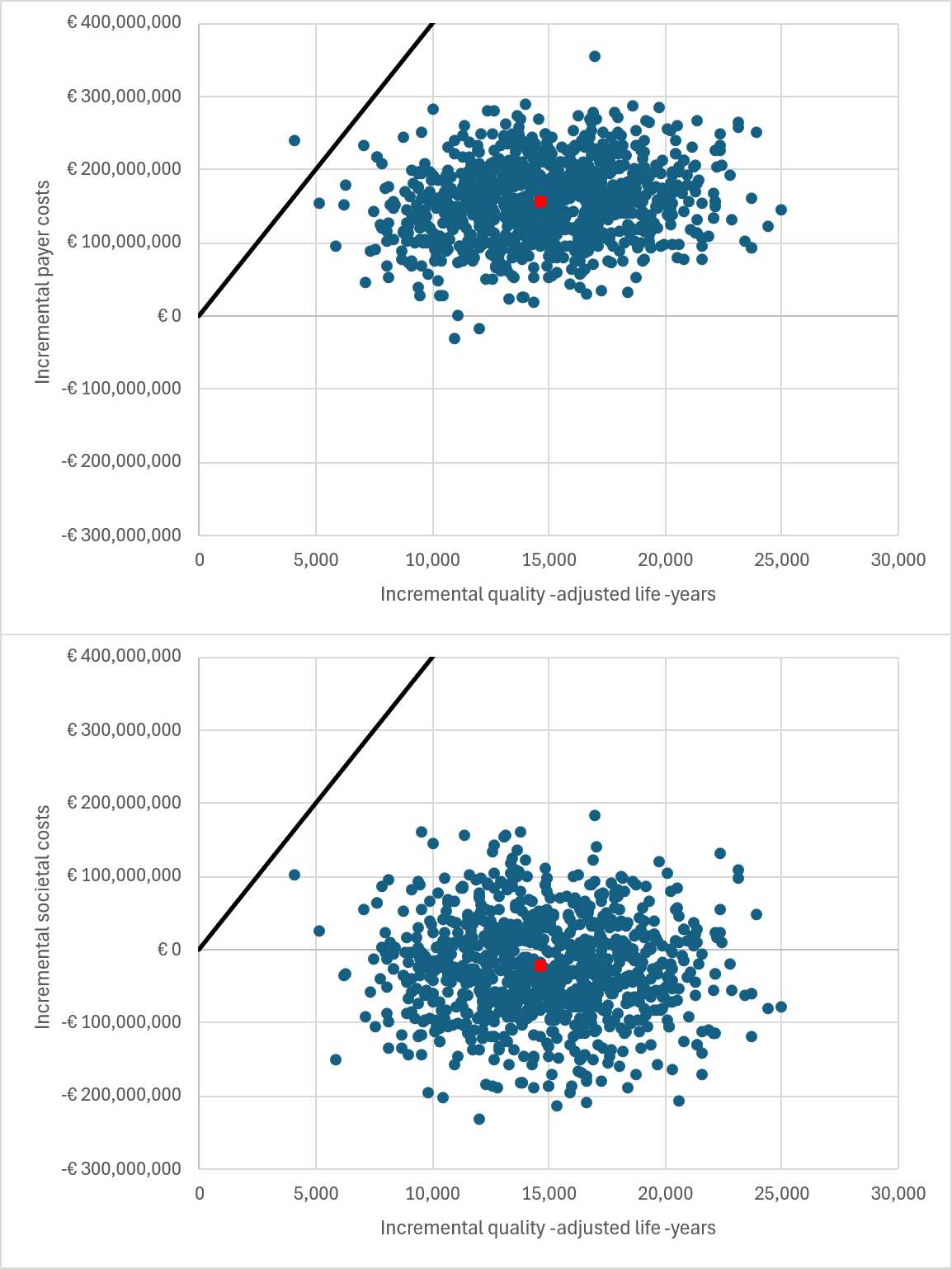


Fig M: Marginal cost versus marginal QALY scatter plot for Strategy 1 probabilistic sensitivity analysis (PSA) results

**(Square) Model outcome with base case values. (Circles) Probabilistic sensitivity analysis realizations. (Line) Willingness-to-pay threshold.**


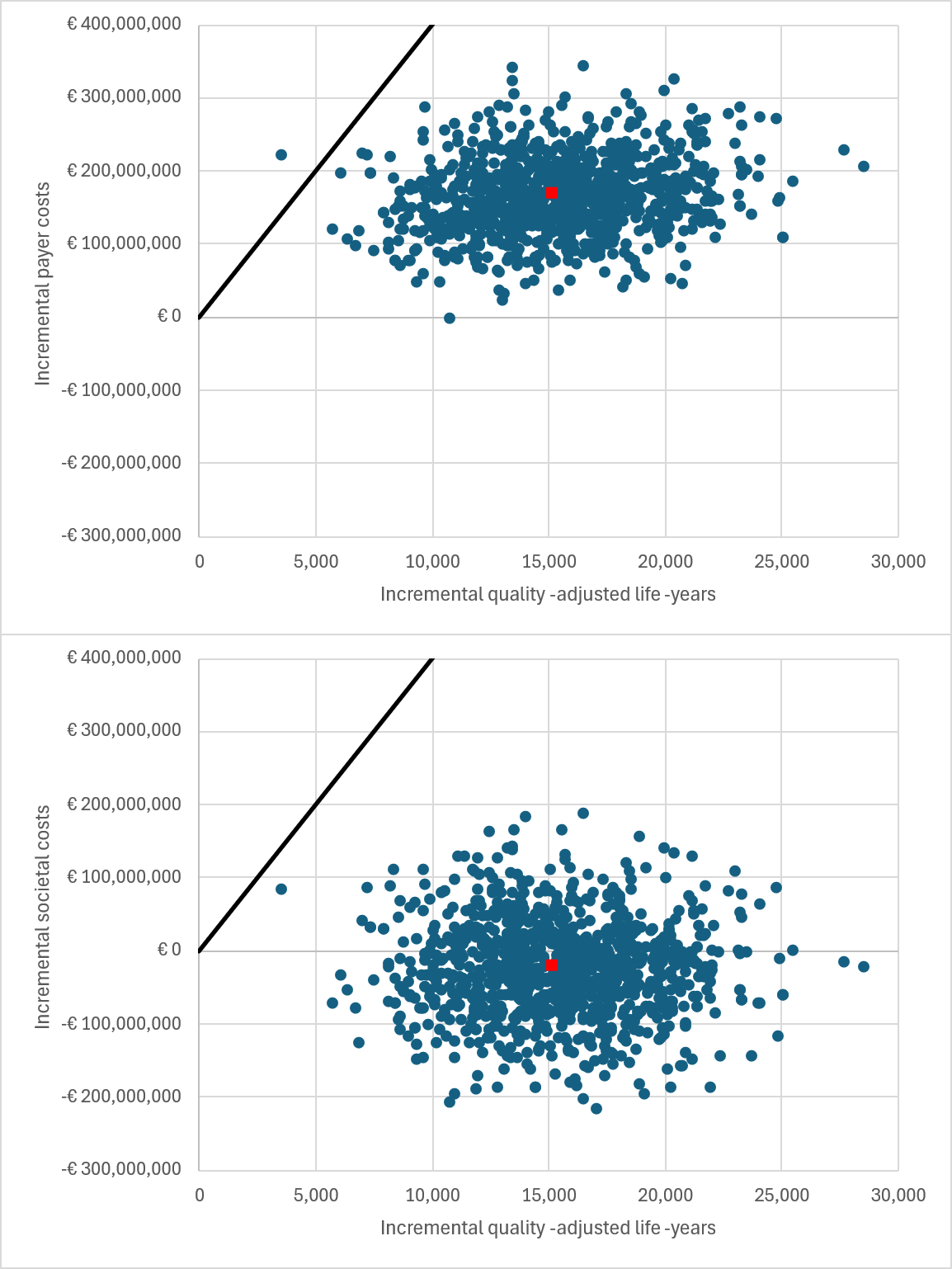


Fig N: Marginal cost versus marginal QALY scatter plot for Strategy 2 probabilistic sensitivity analysis (PSA) results

**(Square) Model outcome with base case values. (Circles) Probabilistic sensitivity analysis realizations. (Line) Willingness-to-pay threshold.**


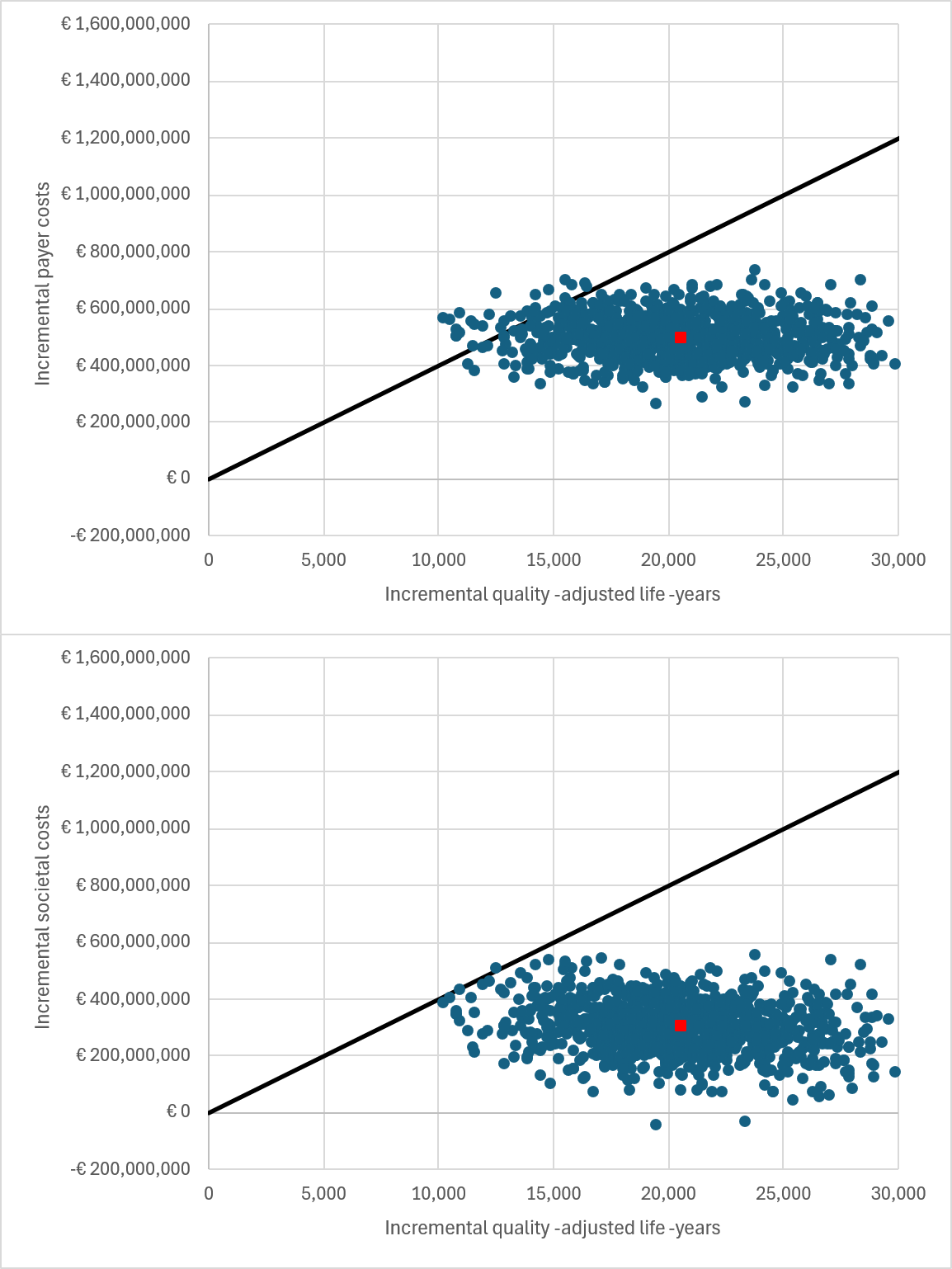


Fig O: Marginal cost versus marginal QALY scatter plot for Strategy 3 probabilistic sensitivity analysis (PSA) results

**(Square) Model outcome with base case values. (Circles) Probabilistic sensitivity analysis realizations. (Line) Willingness-to-pay threshold.**

# References

[1] Statbel. *Population de la Belgique par age, au 1er janvier - Hommes et femmes*. [Online]. Available: <https://statbel.fgov.be/nl>

[2] Statbel. *Quotients de mortalité - Observations - Belgique - Hommes et femmes*. [Online]. Available: <https://statbel.fgov.be/nl>

[3] United Nations Department of Economic and Social Affairs - Population Division. World Population Prospects 2022, Online Edition [Online] Available: <https://population.un.org/wpp/>

[4] Statbel. *Mouvement de la population de la Belgique - Hommes et femmes*. [Online]. Available: <https://statbel.fgov.be/nl>

[5] *DemoTools: An R package of tools for aggregate demographic analysis*. (2019). [Online]. Available: <https://github.com/timriffe/DemoTools/>

[6] A. Rogers and L. J. Castro, *Model Migration Schedules*. Laxenburg, Austria: International Institute for Applied Systems Analysis, 1981.

[7] K. Prem *et al.*, "Projecting contact matrices in 177 geographical regions: An update and comparison with empirical data for the COVID-19 era," *PLOS Computational Biology,* vol. 17, no. 7, p. e1009098, 2021, doi: 10.1371/journal.pcbi.1009098.

[8] O. Sharomi *et al.*, "Modeling the Impact of Exogenous Boosting and Universal Varicella Vaccination on the Clinical and Economic Burden of Varicella and Herpes Zoster in a Dynamic Population for England and Wales," *Vaccines,* vol. 10, no. 9, p. 1416, 2022. [Online]. Available: <https://www.mdpi.com/2076-393X/10/9/1416>.

[9] M. C. Schuette and H. W. Hethcote, "Modeling the effects of varicella vaccination programs on the incidence of chickenpox and shingles," *Bulletin of Mathematical Biology,* vol. 61, no. 6, pp. 1031-1064, 1999/11/01 1999, doi: 10.1006/bulm.1999.0126.

[10] A. A. Gershon, R. Raker, S. Steinberg, B. Topf-Olstein, and L. M. Drusin, "Antibody to Varicella-Zoster Virus in Parturient Women and Their Offspring During the First Year of Life," *Pediatrics,* vol. 58, no. 5, pp. 692-696, 1976, doi: 10.1542/peds.58.5.692.

[11] J. E. Gordon and F. M. Meader, "THE PERIOD OF INFECTIVITY AND SERUM PREVENTION OF CHICKENPOX," *Journal of the American Medical Association,* vol. 93, no. 26, pp. 2013-2015, 1929, doi: 10.1001/jama.1929.02710260001001.

[12] D. L. Heymann, *Control of Communicable Diseases Manual: An Official Report of the American Public Health Association*. Washington, DC, USA: American Public Health Association, 2015.

[13] H. S. Izurieta, P. M. Strebel, and P. A. Blake, "Postlicensure effectiveness of varicella vaccine during an outbreak in a child care center," (in eng), *Jama,* vol. 278, no. 18, pp. 1495-9, Nov 12 1997.

[14] H. Forbes *et al.*, "Risk of herpes zoster after exposure to varicella to explore the exogenous boosting hypothesis: self controlled case series study using UK electronic healthcare data," *BMJ,* vol. 368, p. l6987, 2020, doi: 10.1136/bmj.l6987.

[15] J. F. Seward, J. X. Zhang, T. J. Maupin, L. Mascola, and A. O. Jumaan, "Contagiousness of varicella in vaccinated cases: a household contact study," (in eng), *Jama,* vol. 292, no. 6, pp. 704-8, Aug 11 2004, doi: 10.1001/jama.292.6.704.

[16] P. Poletti *et al.*, "Perspectives on the impact of varicella immunization on herpes zoster. A model-based evaluation from three European countries," (in eng), *PLoS One,* vol. 8, no. 4, p. e60732, 2013, doi: 10.1371/journal.pone.0060732.

[17] J. Trollor, "Herpes zoster in general practice," *Aust. Fam. Physician,* vol. 16, pp. 1137-1140, 1987.

[18] M. Riera-Montes *et al.*, "Estimation of the burden of varicella in Europe before the introduction of universal childhood immunization," *BMC Infectious Diseases,* vol. 17, no. 1, p. 353, 2017/05/18 2017, doi: 10.1186/s12879-017-2445-2.

[19] Z. Pieters, B. Ogunjimi, P. Beutels, and J. Bilcke, "Cost-Effectiveness Analysis of Herpes Zoster Vaccination in 50- to 85-Year-Old Immunocompetent Belgian Cohorts: A Comparison between No Vaccination, the Adjuvanted Subunit Vaccine, and Live-Attenuated Vaccine," *PharmacoEconomics,* vol. 40, no. 4, pp. 461-476, 2022/04/01 2022, doi: 10.1007/s40273-021-01099-2.

[20] J. Mossong *et al.*, "Parvovirus B19 infection in five European countries: seroepidemiology, force of infection and maternal risk of infection," (in eng), *Epidemiol Infect,* vol. 136, no. 8, pp. 1059-68, Aug 2008, doi: 10.1017/s0950268807009661.

[21] J. Bilcke *et al.*, "The health and economic burden of chickenpox and herpes zoster in Belgium," *Epidemiology and Infection,* vol. 140, no. 11, pp. 2096-2109, 2012, doi: 10.1017/S0950268811002640.

[22] C. Truyers, G. Goderis, H. Dewitte, M. v. Akker, and F. Buntinx, "The Intego database: background, methods and basic results of a Flemish general practice-based continuous morbidity registration project," *BMC Medical Informatics and Decision Making,* vol. 14, no. 1, p. 48, 2014/06/06 2014, doi: 10.1186/1472-6947-14-48.

[23] N. Bouckaert, S. Gerkens, S. Devriese, and I. Cleemput, "An EQ-5D-5L value set for Belgium – How to value healthrelated quality of life? ," Health Services Research (HSR) Brussels: Belgian Health Care Knowledge Centre (KCE). 2021.

[24] M. Brisson and W. J. Edmunds, "Varicella vaccination in England and Wales: cost-utility analysis," *Archives of Disease in Childhood,* vol. 88, no. 10, pp. 862-869, 2003, doi: 10.1136/adc.88.10.862.

[25] A. J. van Hoek, N. Gay, A. Melegaro, W. Opstelten, and W. J. Edmunds, "Estimating the cost-effectiveness of vaccination against herpes zoster in England and Wales," *Vaccine,* vol. 27, no. 9, pp. 1454-1467, 2009/02/25/ 2009, doi: <https://doi.org/10.1016/j.vaccine.2008.12.024>.

[26] A. Gauthier, J. Breuer, D. Carrington, M. Martin, and V. RÉMy, "Epidemiology and cost of herpes zoster and post-herpetic neuralgia in the United Kingdom," *Epidemiology and Infection,* vol. 137, no. 1, pp. 38-47, 2009, doi: 10.1017/S0950268808000678.

[27] Belgian Center for Pharmacotherapeutic Information. "Vaccin tegen zona." <https://www.bcfi.be/nl/chapters/13?frag=20636> (accessed 03/21/2023.

[28] J. Carrico *et al.*, "Public health impact and return on investment of Belgium’s pediatric immunization program," (in English), *Frontiers in Public Health,* Original Research vol. 11, 2023-June-22 2023, doi: 10.3389/fpubh.2023.1032385.

[29] F. Zhou, I. R. Ortega-Sanchez, D. Guris, A. Shefer, T. Lieu, and J. F. Seward, "An Economic Analysis of the Universal Varicella Vaccination Program in the United States," *The Journal of Infectious Diseases,* vol. 197, no. Supplement_2, pp. S156-S164, 2008, doi: 10.1086/522135.

[30] J. Bilcke *et al.*, "Rapport coût-utilité de la vaccination contre la varicelle chez les enfants, et de la vaccination contre le zona chez les adultes en Belgique. Health Technology Assessment (HTA)," in "KCE Reports," Centre fédéral d’expertise des soins de santé (KCE), Bruxelles, 2010.

[31] StatBel. "An overview of Belgian wages and salaries." <https://statbel.fgov.be/en/themes/work-training/wages-and-labourcost/overview-belgian-wages-and-salaries> (accessed 05/11/2023, 2023).

[32] A. Gater, L. Abetz-Webb, S. Carroll, A. Mannan, M. Serpell, and R. Johnson, "Burden of herpes zoster in the UK: findings from the zoster quality of life (ZQOL) study," *BMC Infectious Diseases,* vol. 14, no. 1, p. 402, 2014/07/20 2014, doi: 10.1186/1471-2334-14-402.

[33] M. Pillsbury, C. Carias, S. Samant, D. Greenberg, and M. Pawaskar, "Comparison of performance of varicella vaccines via infectious disease modeling," *Vaccine,* vol. 40, no. 29, pp. 3954-3962, 2022/06/23/ 2022, doi: <https://doi.org/10.1016/j.vaccine.2022.05.003>.

[34] C. Boutry *et al.*, "The Adjuvanted Recombinant Zoster Vaccine Confers Long-Term Protection Against Herpes Zoster: Interim Results of an Extension Study of the Pivotal Phase 3 Clinical Trials ZOE-50 and ZOE-70," *Clinical Infectious Diseases,* vol. 74, no. 8, pp. 1459-1467, 2021, doi: 10.1093/cid/ciab629.

[35] J. C. Lang *et al.*, "The clinical and economic costs associated with regional disparities in varicella vaccine coverage in Italy over 50 years (2020–2070)," *Scientific Reports,* vol. 14, no. 1, p. 11929, 2024/05/24 2024, doi: 10.1038/s41598-024-60649-8.
